# Improvement of protein tertiary and quaternary structure predictions using the ReFOLD refinement method and the AlphaFold2 recycling process

**DOI:** 10.1093/bioadv/vbad078

**Published:** 2023-06-14

**Authors:** Recep Adiyaman, Nicholas S Edmunds, Ahmet G Genc, Shuaa M A Alharbi, Liam J McGuffin

**Affiliations:** School of Biological Sciences, University of Reading, Reading RG6 6EX, UK; School of Biological Sciences, University of Reading, Reading RG6 6EX, UK; School of Biological Sciences, University of Reading, Reading RG6 6EX, UK; School of Biological Sciences, University of Reading, Reading RG6 6EX, UK; School of Biological Sciences, University of Reading, Reading RG6 6EX, UK

## Abstract

**Motivation:**

The accuracy gap between predicted and experimental structures has been significantly reduced following the development of AlphaFold2 (AF2). However, for many targets, AF2 models still have room for improvement. In previous CASP experiments, highly computationally intensive MD simulation-based methods have been widely used to improve the accuracy of single 3D models. Here, our ReFOLD pipeline was adapted to refine AF2 predictions while maintaining high model accuracy at a modest computational cost. Furthermore, the AF2 recycling process was utilized to improve 3D models by using them as custom template inputs for tertiary and quaternary structure predictions.

**Results:**

According to the Molprobity score, 94% of the generated 3D models by ReFOLD were improved. AF2 recycling showed an improvement rate of 87.5% (using MSAs) and 81.25% (using single sequences) for monomeric AF2 models and 100% (MSA) and 97.8% (single sequence) for monomeric non-AF2 models, as measured by the average change in lDDT. By the same measure, the recycling of multimeric models showed an improvement rate of as much as 80% for AF2-Multimer (AF2M) models and 94% for non-AF2M models.

**Availability and implementation:**

Refinement using AlphaFold2-Multimer recycling is available as part of the MultiFOLD docker package (https://hub.docker.com/r/mcguffin/multifold). The ReFOLD server is available at https://www.reading.ac.uk/bioinf/ReFOLD/ and the modified scripts can be downloaded from https://www.reading.ac.uk/bioinf/downloads/.

**Supplementary information:**

Supplementary data are available at *Bioinformatics Advances* online.

## 1 Introduction

There has been a CASP community effort to predict protein structures at high accuracy for three decades (Kryshtafovych *et al.*, 2019; Senior *et al.*, 2019; Subramaniam and Kleywegt, 2022). At CASP14, DeepMind’s AlphaFold group submitted tertiary structure models which were widely accepted to represent a step-change in model quality. The models from the AlphaFold2 (AF2) method reached near experimental accuracy for computational modeling. Nevertheless, the actual process of the protein folding path, the effect of conformational flexibility and mutations on functionality and interacting partners remain unclear (Marx, 2022; Skolnick *et al.*, 2021).

### 1.1 The role of the molecular dynamics simulations for improving AF2 structure predictions

AF2 predictions were found to be highly accurate in CASP14 (Jumper *et al.*, 2021b; Marx, 2022; Skolnick *et al.*, 2021). However, a downside of AF2 is that it provides only a few 3D models rather than conformational dynamics (Jumper *et al.*, 2021b; Skolnick *et al.*, 2021), and 3D model comparisons based on mostly C-alpha superpositions may not yield sufficient data to comprehend protein functions. The Molecular Dynamics (MD) protocols, which employ physics-based force fields, may rationalize protein interactions and functions by simulating structural conformational changes that may occur under cellular conditions.

AF2 can predict protein structures at high accuracy while also providing accurate local quality estimates. Protein structure prediction methods have traditionally provided per-residue accuracy scores with the model coordinate data (Varadi *et al.*, 2022). AF2 generates a predicted per-residue accuracy score, which is based on lDDT-Cα and ranges from 0 to 100, with higher scores indicating a better prediction for each residue (Jumper *et al.*, 2021b; Marx, 2022; Skolnick *et al.*, 2021).

Since CASP13, we have been pioneering the use of local quality estimates to guide our MD simulation protocols by restraining highly accurate regions and focusing attention on the poorly predicted regions for model refinement. ReFOLD2 was developed by applying a threshold based on the local quality estimation to restrain highly predicted regions in CASP13 (Adiyaman and McGuffin, 2019). In CASP14, the restraint strategy was further developed to apply a gradual restraint rather than a threshold (Adiyaman and McGuffin, 2021). This protocol was made available to the community as the ReFOLD3 web server, which was among the top-performing protocols in CASP14 (Adiyaman and McGuffin, 2021). In previous studies, alternative protocols were also developed for carrying out MD simulations using AF2 models (Arantes *et al.*, 2021; Heo *et al.*, 2021). These MD simulation protocols might be useful for the simulation of biological systems using AF2 models, but they may not always be relied upon to improve the backbone quality of local regions in AF2 models. Although such methods may not cause significant deviations from the AF2 models, they were not specifically designed to refine them (Arantes *et al.*, 2021; Heo *et al.*, 2021). Furthermore, Lim Heo *et al.* (2021) found that their refinement protocol, which was usually successful on other models, often decreased the quality of AF2 models (Heo *et al.*, 2021). Our aim with ReFOLD4 is to refine AF2 models, maintaining high accuracy in local regions, and preventing structural drift, while using modest computational resources compared to traditional MD-based refinement protocols. Here, we have adapted our ReFOLD3 protocol to utilize the AF2 built-in local quality estimates, which are available in the B-factor column in the predicted 3D models.

### 1.2 Using the AF2 custom template option with further recycling to improve the quality of input protein structure models

AF2’s algorithmic model is based on two key processes; multiple sequence alignments (MSA) and deep neural networks (DNN). While neither concept is particularly new to the modeling community, their unique combination, along with the ability to construct detailed residue pair representations appeared to be key to AF2’s success.

There was also a third interesting process; the existence of a recycling route, which allowed repeated passing of the partially completed proto-model through the DNNs until no further improvement was detectable. If the input of models could be controlled, then this represents a ready-made refinement loop.

ColabFold is a publicly available adaptation of AF2 using a fast MMseqs2 search facility (Mirdita *et al.*, 2022), which also includes a custom template function. This can be adapted to add templates directly into the recycling loop. Our hypothesis for using ColabFold in our CASP15 modeling pathway was that the custom template option could be utilized to input full models into the recycling loop for refinement. Support for this viewpoint comes from the ColabFold team’s recent paper; State-of-the-Art Estimation of Protein Model Accuracy using AlphaFold (Roney and Ovchinnikov, 2022).

For protein complexes, traditional rigid-body docking does not allow for any conformational changes on binding. Thus, refinement becomes an essential step if the model is to be used for drug design or protein–protein interactions (Verburgt and Kihara, 2022). Several docking programs already include flexible modeling, such as iATTRACT (Schindler *et al.*, 2015) and HADDOCK (De Vries *et al.*, 2010), in an attempt to improve natural contacts at the interface and provide more native shape conformity (Schindler *et al.*, 2015). The Deepmind group designed AF2_Multimer (Evans *et al.*, 2021) specifically to model complex structures following Yoshitaka Moriwaki’s work showing that AF2 could be used to model complex protein structures using a linker between two chains.

To test our hypothesis, here we selected two sets of CASP14 tertiary and quaternary structure models and used the preCASP14-trained AF2 model (see Methods below) to recycle them. For tertiary structures we chose DeepMind’s AlphaFold group’s (group 427) official CASP14 submissions as well as models selected from the five groups ranking immediately below 427. As AlphaFold did not submit quaternary structures for CASP14, models were generated for the CASP14 targets using AF2-Multimer. Non-AF2 quaternary structure models from the top groups were also used, as described above for monomers.

We controlled for the argument that this procedure amounted to simple remodeling in two ways; firstly, by using the official AlphaFold group’s CASP14 models with the AF2 model trained on pre-CASP14 data—the rationale being that the same software should not be able to improve upon its original model. Secondly, by running parallel MSA and single sequence recycling we controlled for any influence that an updated MSA might introduce. Consistent improvement under these conditions would suggest that AF2 recycling process is indeed refining the baseline models.

## 2 Methods

### 2.1 The ReFOLD protocol

In our modified ReFOLD4 pipeline, we further improved the MD-based protocol of ReFOLD3 to provide the conformational landscape of the AF2 predictions by applying a unique fine-grained restraint strategy. To fix the local errors identified by the built-in local quality estimation, a fine-grained restraint strategy based on the plDDT score was proposed. In other words, the plDDT score was used as the force constant to be multiplied by the weak harmonic positional restraints (0.05 kcal/mol/Å^2^) (Mirjalili *et al.*, 2014) for each residue on all atoms, including C-alphas, during the MD simulation. As a result, each residue's restraint sensitivity varies according to its accuracy score, where the restraint ranges from 0.05 to 5 kcal/mol/Å^2^ instead of determining a local quality estimation score range as used previously in the ReFOLD3 protocol (Adiyaman and McGuffin, 2021).

In our first version of the ReFOLD method (Shuid *et al.*, 2017), the MD simulation parameters were optimized for a modest computational resource compared to other MD-based protocols tested in CASP experiments. The optimized parameters were also used in this MD-based protocol. In summary, the MD simulations were conducted using NAMD version 2.10 via a parallel GPU-based implementation (Phillips *et al.*, 2005). A CHARMM22/27 force field was used to describe the system (Best *et al.*, 2012), the structure was solvated with the TIP3 water model (Jorgensen *et al.*, 1983), and the total charge was neutralized with Na+ or Cl– ions using Particle Mesh Ewald (PME) (Götz *et al.*, 2012). The simulations were performed at 298 K with 1 bar using Langevin dynamics (Loncharich *et al.*, 1992) for temperature and pressure coupling. The default simulation parameters of CHARMM27 were used to exclude non-bonded interactions, which are mostly van der Waal bonds with a switching distance of 10 Å (Mirjalili *et al.*, 2014). Also, hydrogen bonds were rigidified using the rigidBonds function with a 2 fs timestep (Mirjalili *et al.*, 2014). For the first step of MD simulation, an energy minimization protocol for 1000 steps was applied, followed by the main MD simulation. The fine-grained harmonic positional constraints on all atoms including C-alphas were also performed for 2 ns for each of four parallel simulations for a total of 8 ns. Following the completion of the MD simulation, protein images were generated for each 50 ps to generate 164 3D models in total.

LocalColabFold (Mirdita *et al.*, 2022) was used to generate AF2 monomer predictions for CASP14 structures in the default mode (3 recycles) without using the template option, and these 3D models were used as starting models for the refinement pipeline. The best AF2 predictions during CASP14, which were for FM targets, were also further refined using the ReFOLD4 protocol. 16 FM targets were investigated, which had official observed structures available via the CASP website. ReFOLD4 generated 164 3D models for all CASP14 targets, but only 82 3D models were generated for T1096. Therefore, to maintain a consistent number of models for all targets analysed, T1096 was excluded from the ReFOLD4 analysis, since it was the only one that we encountered which produced a lower number.

### 2.2 The AF2 recycling protocol for AlphaFold monomeric models

The CASP14 rank 1 AlphaFold tertiary models were downloaded from the CASP website along with their official scores and observed experimental structures. Previous studies have suggested that models created using highly accurate template-based modeling (TBM) have less room for improvement than those created using free modeling (FM) methods (Adiyaman and McGuffin, 2021). Therefore, to maximize the refinement potential, and to match the ReFOLD4 process above, we used the same 16 CASP14 FM models as starting structures.

Two structural alignment scoring methods were used to provide performance metrics for model benchmarking. These scores, generated by downloadable versions of TM-score (Zhang and Skolnick, 2004) and lDDT score (Mariani *et al.*, 2013), describe the backbone (TM-score) and local environment (lDDT) similarities of predicted and observed protein structures. The ‘baseline’ TM and lDDT scores were obtained by comparing the downloaded starting models for each target from each group with the observed structures.

The model PDB files were then converted to mmCIF format with https://mmcif.pdbj.org/converter using the RSCB PDB MAXIT suite of programs. The converted model files were then submitted to the Google Colaboratory hosted ColabFold [release 3, v1.3.0 (4-Mar-2022)] as custom templates along with their respective amino acid sequences. Each individual model was used to generate two distinct sub-populations of recycled models; those where ColabFold was allowed to generate a multiple sequence alignment (MSA models) and those for which an MSA was not permitted (single-sequence models). The difference between these approaches is based on a single ColabFold parameter option. Within the MSA and single sequence modes each starting model was submitted four times for 1, 3, 6 and 12 recycles. ColabFold settings used were: Template_mode: custom; msa_mode: MMseqs2 (UniRef+Environmental) OR single sequence; pair_mode: unpaired+paired; model-type: auto; num_recycles: 1, 3, 6, 12. (N.B. Selecting ‘auto’ from the model type defaulted to monomer_ptm, which was the original pre-CASP14 model). Amber relaxation was not enabled as a control measure to ensure we were testing for the recycling effect only.

The five models created for each ColabFold run were collected along with their predicted pTM and plDDT scores. Rank 1 models were then rescored with TM-score and lDDT in the same way as described for baseline models. Scores obtained at baseline and for each recycle combination, along with predicted scores (pTM and plDDT), were then directly compared. Statistical analysis was performed using R-studio.

### 2.3 The AF2 recycling protocol for non-AlphaFold monomeric models

The same 16 CASP14 targets were selected from the next five best-ranked groups beneath AlphaFold at CASP14. These are (by rank): Baker, Baker-experimental (Baek *et al.*, 2021), Feig-R2 (Heo *et al.*, 2021), Zhang (Zheng *et al.*, 2021) and tFold_human (Shen *et al.*, 2021). In addition, to make the test more challenging, only models with a CASP TM-Score of ≥0.45 were used, as those below this threshold cannot be guaranteed to have the same fundamental fold as the reference models (Xu and Zhang, 2010). A total of 47 models were processed.

Models and the observed reference structures for these targets were downloaded from the CASP14 website and scored with TM-score and lDDT in the same way as described in Section 2.2. ColabFold recycling using MSA was submitted to the same Google Colaboratory version of ColabFold as referenced in Section 2.2, recycling using single sequence mode was carried out using release v1.3.0 of Localcolabfold (Mirdita *et al.*, 2022) installed on our own server, to overcome the Google Colaboratory GPU restrictions in the time available. The equivalent Localcolabfold settings were used: msa-mode: single_sequence; model-type: auto; rank: plddt; pair-mode: unpaired+paired; templates: –custom-template-path.

### 2.4 The AF2M recycling protocol for multimeric models

We repeated the process described above but this time using the multimeric CASP14 targets. Ten CASP14 targets were used (H1045, H1065, H1072, T1032, T1054, T1070, T1073, T1078, T1083, T1084) for which the top 5 performing groups had all provided quaternary structure predictions [Baker-experimental (Baek *et al.*, 2021), Venclovas (Dapkūnas *et al.*, 2021), Takeda-Shitaka, Seok (Park *et al.*, 2021) and DATE]. Since the AF group did not submit any models for multimeric targets in CASP14, we also generated AF2-Multimer (AF2M) models for the same targets, so we could then perform common subset analysis. Despite the use of the multimer structures generated in CASP14 as templates, AlphaFold2-Multimer utilizes monomeric structures as templates by dividing the multimeric structures into individual chains.

Each of the baseline models was then refined using the template option and similar parameters (for both MSA and single_sequence options) as described above for monomers. We also aimed to establish the optimal number of recycles for complex structures. The default value for monomeric AF2 structures is three recycles, however we also tested out several different numbers of recycles for the multimers. For scoring the modeled complexes, the MM-Align (Mukherjee and Zhang, 2009) and OpenStructure programs were used for obtaining observed scores for the TM-score and the oligo IDDT score respectively. In addition, we used the QS-score from OpenStructure which considers the interfaces of assemblies. The QS-score is useful for the comparison of homo- and hetero-complexes with different stoichiometries, various orientations of relative chains and varied amino acid sequences (Bertoni *et al.*, 2017).

## 3 Results and discussion

### 3.1 The performance of the ReFOLD pipeline

We used our new ReFOLD4 protocol to refine 57 regular CASP14 targets predicted by LocalColabFold. Out of the 57 targets, 29 were designated as TBM, 15 as FM and 13 as FM/TBM. The 15 FM top predictions made by the AF2 group (427) during CASP14 were also further refined to test ReFOLD4's capabilities. C-alpha-based assessment scores such as GDT-TS (Zhang and Skolnick, 2004) and lDDT (Mariani *et al.*, 2013) were also used alongside the Molprobity score to evaluate ReFOLD4's performance (Adiyaman and McGuffin, 2019).

In contrast to the GDT-TS score, Molprobity (Chen *et al.*, 2010) takes into account all atoms rather than just C-alpha superpositions as a non-native structure-dependent scoring method. Experimental structures may also be determined at low resolution and may contain some local errors, so the Molprobity score may provide an alternative benchmark by reporting atomic clashes, poor rotamers and Ramachandran outliers (Chen *et al.*, 2010). It is remarkable to note that on average, 94% of the 3D models generated by ReFOLD4 were improved compared to the starting models, and all generated models were improved for 46 targets out of 57 targets according to the Molprobity score (see Supplementary Table S1). In addition, ReFOLD4 also improved upon the top models from the AF2 group (427) submitted for CASP14 (∑Molprobitymin = 34.95 and ∑Molprobitymean = 55.86 versus ∑MolprobityAF2CASP14 = 62.91, where lower Molprobity scores are better) (Supplementary Table S1).

Our challenge was also to refine the top-submitted models by AF2 group during CASP14 for 15 FM targets to test the fine-grained restraint strategy's capabilities. It is promising that the fine-grained strategy managed to restrain MD simulations to avoid significant structural deviations according to the GDT-TS and lDDT scores (∑GDTTSmean = 11.35 versus ∑GDTTSstarting = 11.60 and ∑lDDTmean = 10.56 versus ∑lDDTstarting = 11.1) (Supplementary Table S2). A marginal improvement in the overall quality of the top-submitted 3D models was also observed according to the GDT-TS score (∑GDTTSmax = 11.63 versus ∑GDTTSstarting = 11.60) (Supplementary Table S2). ReFOLD4 also showed remarkable success in improving the top-submitted 3D models. Around 72 per cent of the 3D models generated by ReFOLD4 were improved and the cumulative mean and minimum Molprobilty scores were significantly lower than the cumulative Molprobity score of the top-submitted model by AF2 during CASP14 (Supplementary Table S3).

It is assumed that the ReFOLD4 protocol may perform better on models for FM targets, where there is often more room for improvement. ReFOLD4 was able to produce a larger population (>10%) of improved models for 7 out of the 15 targets for the refinement of the starting models generated by LocalColabFold, according to the GDT-TS score (Supplementary Table S4). This indicates that ReFOLD4 can generate a sizable portion of improved models for FM targets. The cumulative maximum GDT-TS score was slightly higher than the cumulative GDT-TS score of the starting models (∑GDT-TSmax = 10.80 versus ∑GDT-TSstarting = 10.75) while the cumulative mean GDT-TS score was slightly lower than the cumulative GDT-TS score of the starting models (∑GDT-TSstarting = 10.75 versus ∑GDT-TSmean = 10.56) (Supplementary Table S4). The higher maximum cumulative GDT-TS score means that ReFOLD4 improved the starting models. A similar trend was observed for the FM/TBM targets.

Historically, the refinement of TBM targets has been more challenging, where models are more likely to deteriorate in global quality following refinement, as there is much less room for improvement (Adiyaman and McGuffin, 2019). The role of the restraint strategy also becomes more apparent in TBM targets, as unrestrained MD simulations are likely to substantially deviate from the native basin (Adiyaman and McGuffin, 2019, 2021). Although there is much less improvement in the overall quality of the 3D models (∑GDT-TSmax = 23.62 versus ∑GDT-TSstarting = 23.49) on 29 TBM targets, the fine-grained restraint strategy managed to direct the generation of 3D models toward the native basin according to the GDT-TS score (∑GDT-TSstarting = 23.49 versus ∑GDT-TSmean = 23.17) as in Supplementary Table S4. It is worth noting that the majority of 3D models generated by ReFOLD4 were improved for T1060s3, T1092, T1093, T1094 and T1095 (Supplementary Fig. S1, and Supplementary Table S4).

The ReFOLD4 pipeline, which used models generated by LocalColabfold as starting models, provided better predictions for 18 targets out of 57 targets (T1030, T1034, T1039, T1046s2, T1047s1, T1047s2, T1049, T1053, T1055, T1061, T1068, T1070, T1078, T1082, T1083, T1084, T1089 and T1095) compared to the AF2 group's (427) top submissions according to the maximum GDT-TS score (Supplementary Table S4).

The lDDT score (Mariani *et al.*, 2013) which is a native structure-dependent score, was also used to analyse the performance of the ReFOLD4 pipeline. Based on this score, ReFOLD4 did not manage to improve most of the targets. Nevertheless, the fine-grained restraint strategy demonstrated exceptional performance for T1092 and T1095 according to the population of the improved models (Supplementary Table S5). The AF2 method is extremely well-trained to optimize the lDDT score, which is the main target measurement for the training dataset (Jumper *et al.*, 2021b; Skolnick *et al.*, 2021). Therefore, further improvements in the overall lDDT quality might not be as achievable by MD simulations. However, significant improvements in lDDT scores for baseline models can be achieved by using the AF2 recycling process for refinement (see below).

### 3.2 Performance of the AF2 recycling protocol for refining monomers

According to the average change in lDDT, monomeric recycling showed an improvement rate of 87.5% (using MSAs) and 81.25% (using single sequences) for AF2 models and 100% (MSA) and 97.8% (single sequence) for non-AF2 models.

A comparison of the improvements in recycled models versus the 16 CASP14 AF2 and 47 non-AF tertiary structure models is presented in Supplementary Table S6. Calculated *P*-values were computed from a 1-tailed Wilcoxon signed-rank test for lDDT scores (Supplementary Table S6A) between the baseline values and the output models from each recycle, and for output models between consecutive recycles. Identical analyses for the TM-scores are presented in Supplementary Table S6B. A significant difference between any two populations is established by a *P*-value of ≤0.05 suggesting an improvement in quality at the given number of recycles.

For lDDT score, given the 0.05 *P*-value threshold, it can be concluded that recycling significantly improved AF2 model quality compared to the baseline models using MSA and single sequence inputs (values shown in bold), although the improvement in model quality was nonlinear; higher recycle numbers did not always show greater improvement.

Further, recycling significantly improved non-AF model quality compared to baseline across all recycles for both MSA and single sequence methods. Improvement in model quality again appeared to be nonlinear; recycle 6–12 produced no further significant improvement for either method (Supplementary Table S6).

Identification of the optimal recycle number was not immediately obvious from the data. However, considering the minimal improvement from recycle 3 onwards for non-AF models and the increasing magnitude of the calculated *P*-values from three recycles onwards for AF2 models, it is reasonable to conclude that three recycles represented a significant improvement at least as good as any other recycle. Therefore, for this population of monomers we will describe recycle 3 as the optimal number of recycles for refinement of monomeric models in agreement with the default value (see Section 2.4).

**Figure 1.**
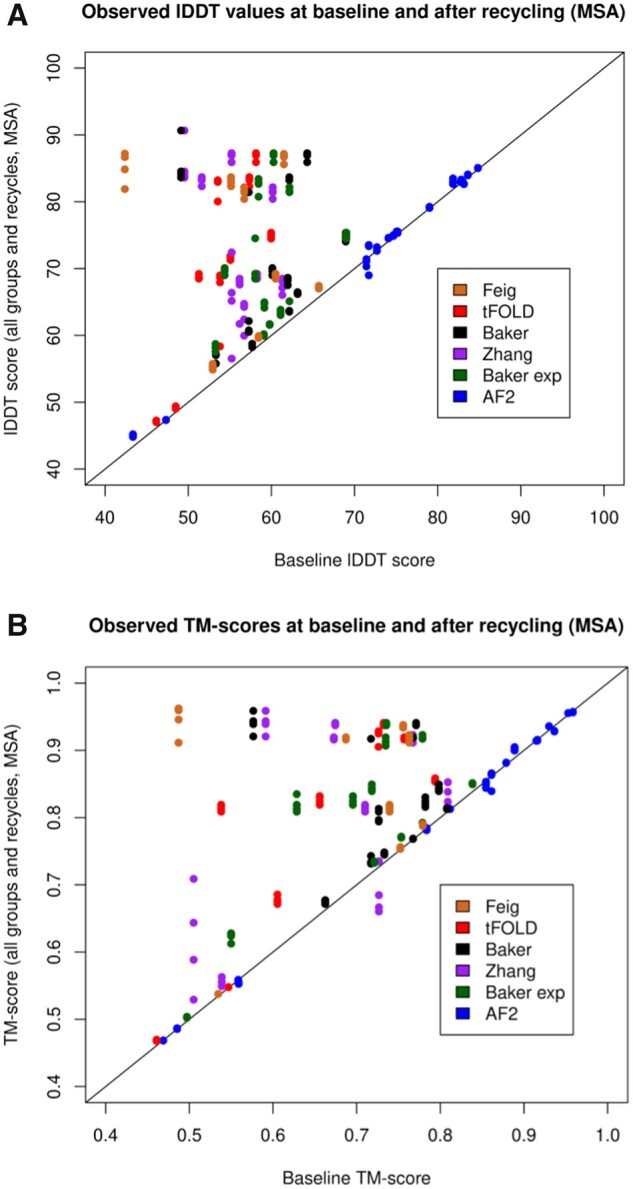
Scatter plots showing the comparison in observed lDDT scores (**A**) and observed TM-scores (**B**) between baseline models (*x*-axis) and models from all recycles (*y*-axis) for all AF2 and non-AF2 models coloured by group (MSA mode recycling)

Figure 1 shows the change in individual model quality for lDDT (Fig. 1A) and TM-score (Fig. 1B) for all models (MSA recycling) with points coloured by group. Comparison plots for three recycles and both recycling modes (MSA and single sequence) are shown in Supplementary Figure S2.

Considering lDDT scores, Figure 1A shows that the improvement of AF2 models (blue points), although significant, was relatively minor in real terms. This is unsurprising considering the high baseline lDDT scores are associated with the AF2 top models and in fact, expected, considering that refinement is associated with small improvements in atomic positions of an already representative model. As explained in the introduction, it is assumed unlikely that any significant remodeling occurred when using official AlphaFold CASP14 structures coupled with the pre-CASP14-trained AF2 model, as the same software should not be able to improve upon its original model without additional information. It is therefore likely that this observed improvement due to recycling represented true refinement by the AF2 algorithm.

For both panels, Figure 1 shows a more noticeable improvement for non-AF models and this is potentially explained by two factors. Firstly, by the lower starting quality of the baseline template models providing greater potential for improvement but, secondly, the possibility that a certain amount of remodeling was taking place during the recycling. It is therefore necessary to identify the extent of this re-modeling and attempt to demonstrate that significant improvement still occurred by recycling alone. In Section 3.3, we compare single-sequence directly with MSA recycling.

There are three interesting outliers in Figure 1 that correspond to refined Zhang models (purple) for recycle 3, 6 and 12 for target T1042. While each of the models increased in lDDT score with recycling (Fig. 1A), they decreased in TM-score (Fig. 1B). In these cases, changes were made in the overall topology, which decreased the TM-score, in order to correct local loop and helix regions, which simultaneously increased the lDDT score. These changes are illustrated in Supplementary Figure S10.

### 3.3 Improvement in MSA versus single sequence recycling

The 1-tailed Wilcoxon signed-rank test was again used to calculate *P*-values between these two model populations. In addition, a 1-tailed Ansari-Bradley test was used to investigate any significant differences in quartiles to rule out differences occurring in the data unrelated to the mean (*P*-values are available in Supplementary Table S7).

It was found that there was no significant difference in model quality between MSA and single sequence recycling for the AF2 models according to both the Wilcoxon and Ansari tests. However, there was a significant difference, detected by both tests, at every recycle for non-AF models. This supports the conjecture in Section 3.1, that no significant remodeling occurred for AF2 models but that a certain amount probably occurred for non-AF models with MSA recycling. The difference between the two graphs may represent the additional remodeling afforded by the MSA for non-AF groups (shown in an equivalent plot in Supplementary Fig. S3).

**Figure 2.**
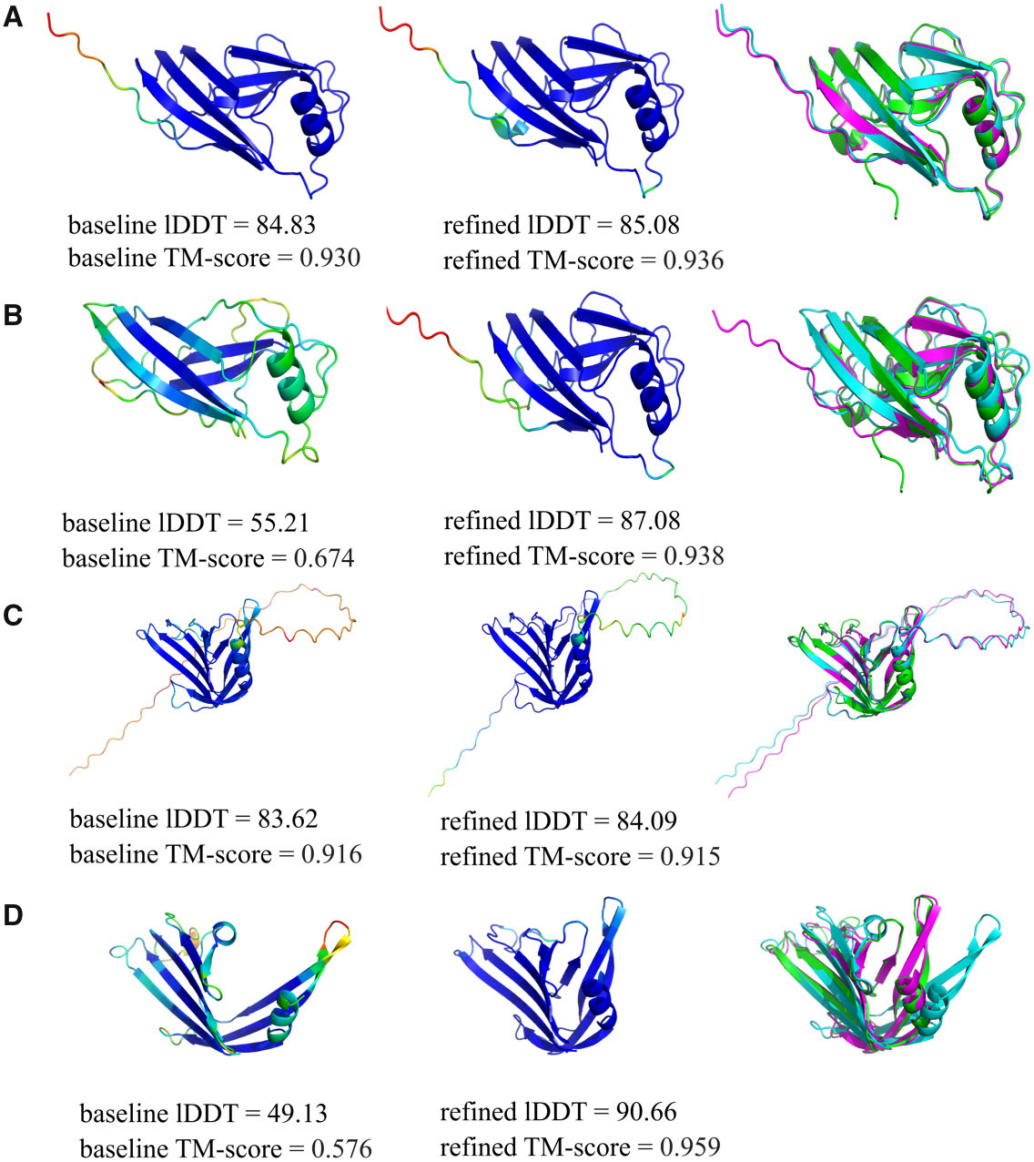
Comparison of monomer models. Images in the left columns show the baseline models coloured by plDDT score. The middle columns show the refined models coloured by plDDT score. The right columns show the superposition of the baseline models (cyan), the refined models (magenta) and the native structures (green). (**A**) AF2 model for T1049: baseline lDDT = 84.83, TM-score = 0.930; refined lDDT = 85.08, TM-score = 0.936. (**B**) Zhang group model for T1049: baseline lDDT = 55.21, TM-score = 0.674; refined lDDT = 87.08, TM-score = 0.938. (**C**) AF2 model for T1074: baseline lDDT = 83.62, TM-score = 0.916; refined lDDT = 84.09, TM-score = 0.915. (**D**) Baker group model for T1074: baseline lDDT = 49.13, TM-score = 0.576; refined lDDT = 90.66, TM-score = 0.959. Images were rendered using PyMOL

Actual changes in lDDT are represented as bar charts in Supplementary Figures S4A and S5. Supplementary Figure S4A shows the change in cumulative lDDT for all groups across all MSA recycles, and the changes in TM-scores are shown in 4B. Both panels clearly show the differences in improvement in model quality obtained between AF2 (blue) and non-AF models (brown, red, black, purple and green columns), again highlighting the differential effect MSA has on the two groups of models. An additional important observation was that many non-AF models with relatively low initial lDDT scores were refined to a level that out-performed both the initial and the refined equivalent AF2 models. Examples of these are shown in Figure 2.

### 3.4 Performance of the AF2M recycling protocol for refining multimers

According to lDDT scores, recycling of multimer models showed an improvement rate of 80% (using MSAs) and 30% (using single sequences) for AF2M models, while 94% (MSA) and 64% (single sequence) non-AF2M models were improved. According to TM-scores, there was an improvement of 70% (MSA) and 80% (single sequence) for AF2M models, and 98% (MSA) and 82% (single sequence) for non-AF2M models. While, according to QS-scores, 50% (MSA) and 30% (single sequence) of AF2M models, and 86% (MSA) and 60% (single sequence) of non-AF2M models were improved.

Compared to the baseline non-AF2 models, significant improvements were made for all numbers of recycles for all scores when MSAs were used. Furthermore, significant improvements to the both TM-score and QS-scores for non-AF2M models were seen for all recycles when only a single sequence was used. While the principal improvement from recycling monomers is evidently the significant gain in lDDT scores, this is not always the case for multimeric models. The AF2M models were more consistently significantly improved in terms of their IDDT-scores, rather than the other scores. Considering the *P*-values for all models and scores, both 6 and 12 recycles appear to be optimal parameters for significantly improving upon baseline models (Supplementary Table S8).

**Figure 3.**
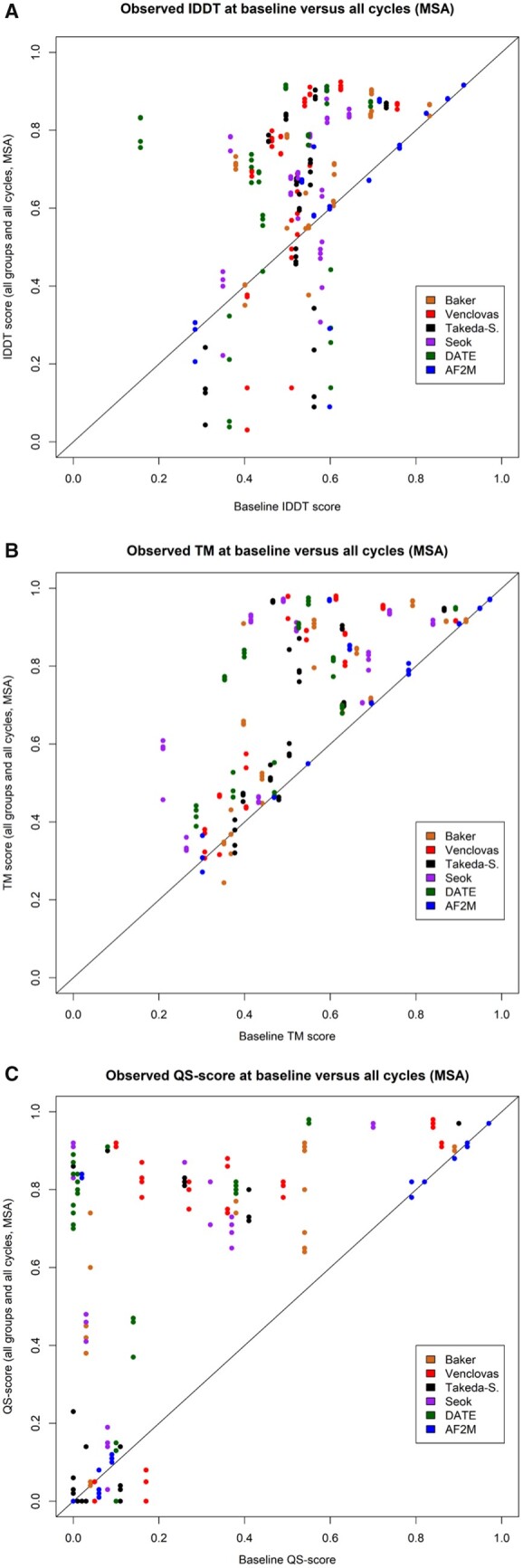
Scatter plots showing the comparison in observed oligo-lDDT (**A**), observed TM (**B**) and observed QS scores (**C**) between baseline (*x*-axis) and all recycles (*y*-axis) for all AF2M and non-AF2M models (MSA mode recycling)

The plots in Figure 3 show that the majority models are improved according to all scoring methods, but again it is clear that the improvement is less consistent for the oligo-lDDT scores (Fig. 3A) than for the TM-scores (Fig. 3B) and QS-scores (Fig. 3C). In Figure 3B, the improvement of TM score for both AF2M and non-AF2M is clearer, and far fewer targets were degraded following increases in the recycling number. Similar plots are shown for each recycle separately in Supplementary Figure S6 and plots for single sequence inputs in Supplementary Figures S7 and S8.

Supplementary Figure S9 shows that the cumulative improvement in model quality was nonlinear with increasing numbers of recycles; higher recycle numbers (>3) did not necessarily always lead to greater improvement for all model types according to all scores. However, with 6–12 recycles models from almost all groups can be improved, with clear cumulative gains shown for each score. While the lDDT and TM scores were improved for all groups, the QS-scores showed the greatest improvements for all recycles compared with the baseline models.

**Figure 4.**
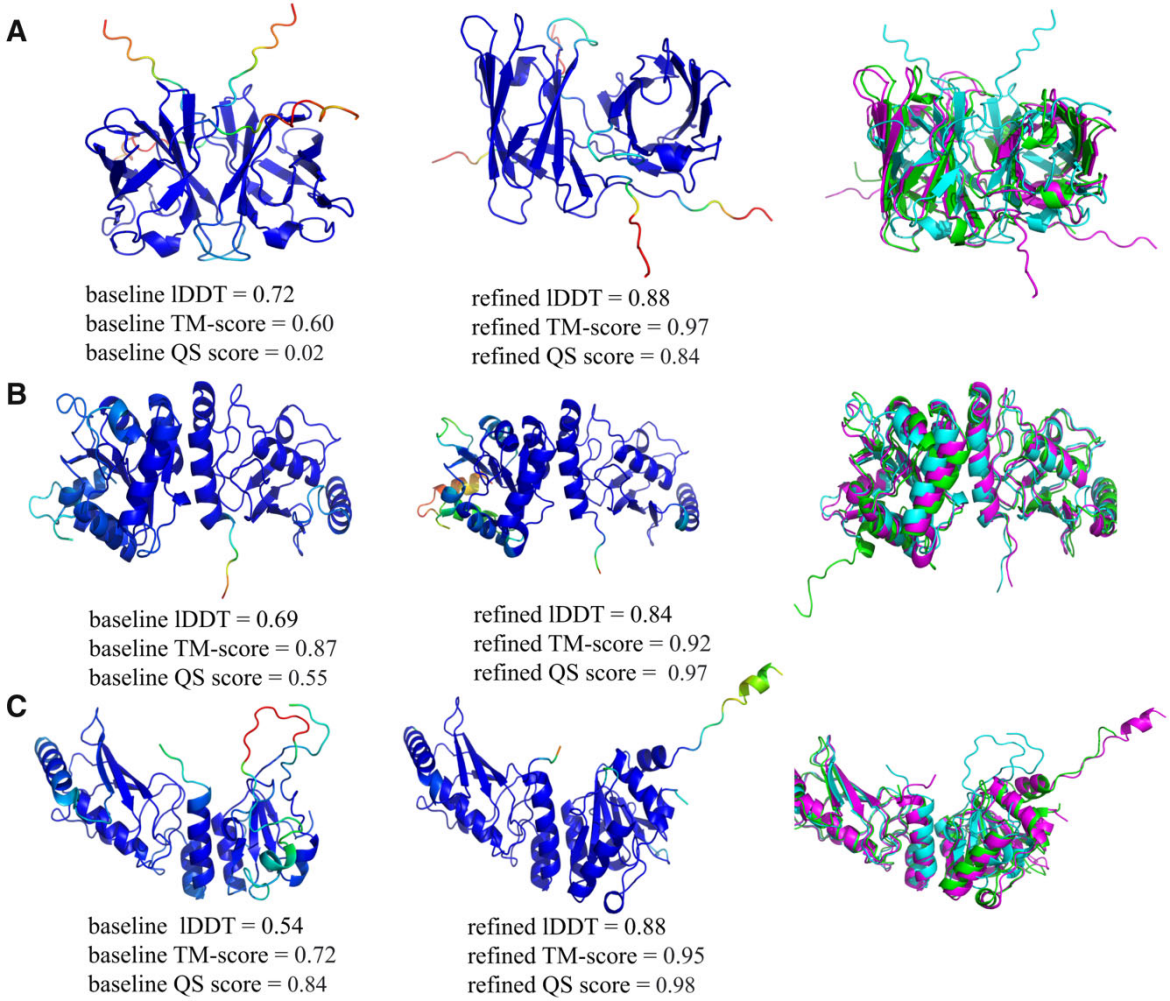
Comparison of multimeric models. Images in the left columns show the starting models coloured by plDDT score. The middle columns show the refined models coloured by plDDT score. The right columns show the superposition of starting models (cyan), the best-refined models generated by colabfold (magenta) and the observed models (green). (**A**) AF2M model for T1078: baseline lDDT = 0.72, TM-score = 0.60, QS score = 0.02; refined lDDT = 0.88, TM-score = 0.97, QS score = 0.84. (**B**) Baker group model for H1045: baseline lDDT = 0.69, TM-score = 0.87, QS score = 0.55; refined lDDT = 0.84, TM-score = 0.92, QS score = 0.97. (**C**) Venclovas group model for H1045: baseline lDDT = 0.54, TM-score = 0.72, QS score = 0.84; refined lDDT = 0.88, TM-score = 0.95, QS score = 0.98. Images were rendered using PyMOL

Figure 4 shows examples of the visible improvements to the quality of multimeric models from different groups following the recycling process. The difference in performance between monomer and complex recycling experiments; namely that monomers show a greater improvement in quality when measured by the lDDT metric whereas complexes tend to show greater improvement when measured by TM-score, is likely a consequence of the different focus of calibration used in the development of the two AF2 versions. AlphaFold2 is primarily calibrated to lDDT (Jumper *et al.*, 2021a; Tunyasuvunakool *et al.*, 2021) whereas AlphaFold2-Multimer is calibrated to TM-Score (Evans *et al.*, 2021). Interestingly, we occasionally observed improvements which appear like remodeling rather than more subtle refinement (Fig. 4A). The quality of the input structure is crucial, and each generated structure serves as a guide for the next recycle. If the generated structure deviates far from the original input, it may result in a remodeled structure for the next recycle.

## 4 Conclusions

We have demonstrated that ReFOLD4 is successful in its main goal of preventing MD simulations from structural deviations and is a risk-averse method for providing the conformational landscape of AF2 accuracy level predictions for further studies, such as drug discovery. It is also promising that by generating a higher population of improved models on FM targets, ReFOLD4 may be of use in de novo protein design pipelines. Molprobity score analysis showed that 94% of models generated by ReFOLD4 were improved, and all models were improved for 46 targets out of 57 compared to the starting models generated by LocalColabFold. 72% of the models were also improved when the top AF2 submissions were used as starting models. It is also worthy of note that our refinement pipeline managed to provide better-improved models for 18 out of 57 targets compared to the AlphaFold group (427) according to the maximum GDT-TS score. The selection of ReFOLD4's best-improved model remains a challenge, despite its high number of improved models.

Furthermore, we have demonstrated that the AF2 recycling process can refine 3D models when they are used as custom template inputs. If the main aim is to consistently improve the quality of a single model, either mono- or multi-meric, then the AF2 recycling process is advantageous in terms of the relatively low computational resources required and it provides a ranking of models for easy selection by predicted quality score (e.g. plDDT or pTM). Both MSA and single sequence input recycling led to a significant improvement in output model quality compared to baseline input models. Importantly, this improvement occurred not only with non-AF models but with original AF2 models that were submitted by DeepMind in CASP14. The lack of significant improvement in consecutive recycles showed that a higher recycle number did not necessarily lead to greater improvement. Comparing the *P*-values, recycle 3 appeared to improve model quality as much as any other recycle and therefore committing monomeric structures to more than three recycles may represent an unnecessary processing overhead. This is shown in Figure 3 by a change in cumulative observed lDDT score of 7.54 for recycle 3 compared to 6.34 at recycle 12 (AF2 models) and a modest 2.7% increase in cumulative observed lDDT score across all non-AF models from recycle 3 to 12. However, for multimeric models, instead of using a recycle value of 3, further recycling appears to be required and, as a general rule, recycle 12 in MSA mode was most successful overall, as measured by IDDT and QS-score.

Molprobity scores were also collected for AF2 recycling (see Supplementary Table S9) but, due to the unrelaxed nature of the recycled models (Amber was not employed), these scores increased suggesting that some unrealistic atomic positions were introduced during the recycling process. Therefore, according to Molprobity scores ReFOLD4 performed better than AF2 recycling for the CASP14 dataset. However, AF2 recycling demonstrated significantly better performance based on the native structure dependent scores (TM and lDDT). As such, a combined approach to refinement using both the ReFOLD4 protocol and the AF2 recycling processes would provide a more complete platform for improving models for further studies, such as drug discovery. The combined approach provides substantial improvements to 3D models beyond the AF2 accuracy level, while requiring modest additional computational resources.

The methods described in this article were deployed in the recent CASP15 experiment for our manual (McGuffin group) prediction pipelines for both multimeric and regular targets. As a result, we were the top-performing academic group from the UK, ranking 6th out of all groups on regular targets and 11th in the multimeric target prediction category. Furthermore, our MultiFOLD server, which also implements the AF2 recycling process as a key part of its pipeline, ranked as the 9th best server group on monomeric targets and the 8th best server group for multimers according to the CASP15 rankings. Notably, MultiFOLD outperformed the baseline NBIS-AF2-standard (AlphaFold2) and NBIS-AF2-multimer (AlphaFold2-Multimer) methods along with many other servers and human predictors (https://predictioncenter.org/casp15/).

## Supplementary Material

vbad078_Supplementary_Data

## Data Availability

The data underlying this article are available in the article, in its online supplementary material and at https://predictioncenter.org/casp15.
