# Supplementary material for: Improvement of protein tertiary and quaternary structure predictions using the ReFOLD refinement method and the AlphaFold2 recycling process
Source: Bioinform Adv. 2023 Jun 14;3(1):vbad078. doi: 10.1093/bioadv/vbad078 (PMC10290552; doi:10.1093/bioadv/vbad078)
Supplement: vbad078_Supplementary_Data [file vbad078_supplementary_data.pdf]

Supplementary Table 1. Performance summary for ReFOLD4 on the CASP14 targets according to Molprobity score (lower Molprobity scores are better). The starting 3D models generated by LocalColabFold were further refined by ReFOLD4.

Target ID	Prediction Method	Score of top model submitted by AF2 in CASP14	Score of starting model generated by ColabFold	Minimum score of refined models	Mean score of refined models	Maximum score of refined models	Percentage of improved models
T1027	FM	2.39	1.16	0.68	1.1432	1.58	63.414
T1029	FM	0.79	1.74	0.5	0.883	1.41	100
T1031	FM	1.62	1.41	0.54	0.985	1.58	98.170
T1033	FM	1.51	1.55	0.51	0.920	1.39	100
T1037	FM	0.9	1.33	0.73	1.080	1.32	100
T1039	FM	1.84	0.88	0.5	0.982	1.34	27.439
T1040	FM	0.5	1.75	0.5	0.842	1.36	100
T1041	FM	1	1	0.52	0.937	1.28	73.780
T1042	FM	1.35	3.66	0.76	1.042	1.33	100
T1043	FM	1.2	1.28	0.52	1.002	1.52	96.341
T1047s1	FM	1.61	1.35	0.77	1.101	1.48	93.292
T1049	FM	0.56	0.89	0.8	1.157	1.58	12.195
T1064	FM	1.14	1.51	0.5	1.125	1.57	98.170
T1074	FM	1.14	1.84	0.97	1.229	1.81	100
T1090	FM	0.68	0.5	0.5	0.899	1.38	0
T1035	FM/TBM	1.02	2.35	0.5	0.764	1.32	100
T1038	FM/TBM	0.83	2.56	0.5	0.972	1.8	100
T1046s1	FM/TBM	1.03	2.3	0.5	0.771	1.3	100
T1047s2	FM/TBM	0.79	2.47	0.71	1.021	1.26	100
T1052	FM/TBM	1.07	2.26	0.78	1.161	1.38	100
T1053	FM/TBM	1.01	2.1	0.71	1.117	1.34	100

T1055	FM/TBM	1.19	2.3	0.5	0.7148	1.2	99.393
T1058	FM/TBM	0.83	1.88	0.53	0.909	1.26	100
T1061	FM/TBM	1.16	2.15	0.78	1.120	1.35	100
T1065s2	FM/TBM	1.19	1.68	0.5	0.831	1.33	100
T1070	FM/TBM	1.41	2.95	0.89	1.268	1.53	100
T1082	FM/TBM	1.22	2.79	0.5	0.919	1.39	100
T1093	FM/TBM	0.9	2.12	0.55	0.812	1.09	100
T1094	FM/TBM	1.18	2.4	0.72	1.011	1.31	100
T1024	TBM	1.06	2.17	0.5	0.795	1.11	100
T1026	TBM	1.83	3.13	0.59	1.162	1.83	100
T1030	TBM	0.72	1.63	0.5	1.188	0.97	100
T1032	TBM	0.66	1.77	0.5	0.844	1.18	100
T1034	TBM	1.43	2.23	0.5	0.939	1.31	100
T1045s2	TBM	1.11	1.81	0.5	0.723	1.11	100
T1046s2	TBM	1.56	2.08	0.53	1.00	1.56	100
T1050	TBM	1.06	1.91	0.68	1.04	1.27	100
T1054	TBM	1.53	2.67	0.9	1.195	1.57	100
T1056	TBM	1.07	2.72	0.84	1.14	1.54	100
T1060s2	TBM	1.18	2.27	0.5	1.018	1.52	100
T1060s3	TBM	0.67	2.27	0.5	1.03	1.54	100
T1065s1	TBM	0.5	1.96	0.5	0.811	1.38	100
T1067	TBM	0.79	2.49	0.79	1.068	1.37	100
T1068	TBM	1.1	2.4	0.58	0.958	1.34	100
T1073	TBM	1.1	2.24	0.5	0.774	1.26	100
T1076	TBM	0.88	1.78	0.5	1.0738	1.37	100
T1078	TBM	1.43	2.38	0.5	0.989	1.41	100
T1079	TBM	0.87	2.25	0.87	1.068	1.35	100

T1083	TBM	0.71	1.78	0.5	0.704	1.22	100
T1084	TBM	0.74	2.02	0.5	0.579	1.11	100
T1087	TBM	0.99	2.65	0.5	0.935	1.55	100
T1089	TBM	1.24	2.39	0.96	1.248	1.51	100
T1092	TBM	0.88	2.17	0.58	0.893	1.17	100
T1095	TBM	1.12	2.25	0.77	0.956	1.26	100
T1099	TBM	1.49	2.96	0.76	1.119	1.45	100
T1100	TBM	1.11	1.85	0.5	0.821	1.17	100
T1101	TBM	1.02	2.39	0.63	1.030	1.4	100
The Cumulative scores		62.91	116.78	34.95	55.86	78.62	

Supplementary Table 2. Performance summary for ReFOLD4 on the AF2 CASP14 FM models according to GDT-TS, and lDDT scores. The top models submitted by AlphaFold2 group (427) were further refined by ReFOLD4.

	GDT-TS score					lDDT score				
CASP Target ID	Score of starting model submitted by AF2 in CASP14	Minimum score of refined models	Mean score of refined models	Maximum score of refined models	Percentage of improved models	Score of starting model submitted by AF2 in CASP14	Minimum score of refined models	Mean score of refined models	Maximum score of refined models	Percentage of improved models
T1027	0.388	0.372	0.382	0.394	12.1951	0.433	0.413	0.418	0.431	0
T1029	0.446	0.428	0.442	0.452	28.658	0.472	0.456	0.461	0.470	0
T1031	0.871	0.828	0.856	0.878	7.317	0.714	0.671	0.687	0.711	0
T1033	0.877	0.832	0.860	0.877	3.658	0.81	0.7513	0.773	0.809	0
T1037	0.872	0.829	0.847	0.873	3.65	0.79	0.744	0.755	0.792	0
T1039	0.823	0.788	0.805	0.818	0	0.71	0.675	0.691	0.714	0
T1040	0.719	0.694	0.713	0.732	23.170	0.74	0.711	0.7303	0.742	4.8788
T1041	0.905	0.862	0.883	0.9081	3.048	0.82	0.76	0.783	0.821	0

T1042	0.837	0.789	0.808	0.838	1.215	0.83	0.77	0.775	0.829	0
T1043	0.832	0.783	0.808	0.831	0	0.75	0.69	0.716	0.743	0
T1047s1	0.502	0.477	0.489	0.5	0	0.75	0.714	0.725	0.745	0
T1049	0.932	0.889	0.9107	0.931	0	0.843	0.780	0.795	0.840	0
T1064	0.803	0.762	0.789	0.811	6.0976	0.746	0.673	0.688	0.733	0
T1074	0.899	0.859	0.878	0.901	3.04878	0.836	0.751	0.770	0.832	0
T1090	0.888	0.863	0.874	0.890	1.2195	0.827	0.778	0.791	0.824	0
The Cumulative scores	11.601	11.063	11.3524	11.638		11.108	10.362	10.56	11.0449	

Supplementary Table 3. Performance summary for ReFOLD4 on the AF2 CASP14 FM models according to Molprobity score. The top models submitted by AlphaFold2 group (427) were further refined by ReFOLD4.

CASP Target ID	Score of starting model submitted by AF2 in CASP14	Minimum score of refined models	Mean score of refined models	Maximum score of refined models	Percentage of improved models
T1027	2.39	0.83	1.217	1.6	100
T1029	0.79	0.5	0.885	1.41	40.243
T1031	1.62	0.5	0.968	1.42	100
T1033	1.51	0.5	0.763	1.16	100
T1037	0.9	0.6	0.9346	1.28	100
T1039	1.84	0.6	0.9215	1.29	100
T1040	0.5	0.48	0.746	1.35	15.853
T1041	1	0.52	0.889	1.24	78.658
T1042	1.35	0.76	1.036	1.31	100
T1043	1.2	0.51	0.950	1.42	90.243
T1047s1	1.61	0.73	1.095	1.49	100
T1049	0.56	0.54	0.9535	1.55	6.097
T1064	1.14	0.5	0.954	1.55	85.365

T1074	1.14	0.81	1.108	1.5	63.414
T1090	0.68	0.52	0.932	1.38	6.097
The Cumulative scores	18.23	8.9	14.357	20.95	

Supplementary Table 4. Performance summary for ReFOLD4 on the CASP14 targets according to GDT-TS score. The starting 3D models generated by LocalColabFold were further refined by ReFOLD4.

CASP Target ID	Prediction Method	Score of top model submitted by AF2 in CASP14	Score of starting model generated by ColabFold	Minimum score of refined models	Mean score of refined models	Maximum score of refined models	Percentage of improved models
T1027	FM	0.388	0.382	0.358	0.368	0.383	4.242
T1029	FM	0.446	0.438	0.42	0.434	0.446	29.878
T1031	FM	0.871	0.844	0.807	0.827	0.844	3.0487
T1033	FM	0.877	0.845	0.812	0.834	0.857	17.073
T1037	FM	0.872	0.729	0.703	0.718	0.729	1.219
T1039	FM	0.823	0.843	0.796	0.8168	0.835	0
T1040	FM	0.719	0.548	0.532	0.546	0.557	45.731
T1041	FM	0.905	0.874	0.848	0.865	0.879	11.585
T1042	FM	0.837	0.633	0.608	0.621	0.635	4.268
T1043	FM	0.832	0.785	0.758	0.779	0.795	25.609
T1047s1	FM	0.502	0.498	0.484	0.494	0.504	17.682
T1049	FM	0.932	0.944	0.906	0.925	0.942	0
T1064	FM	0.803	0.573	0.541	0.562	0.583	10.975
T1074	FM	0.899	0.929	0.884	0.901	0.922	0

T1090	FM	0.888	0.883	0.854	0.871	0.884	2.439
T1035	FM/TBM	0.953	0.887	0.845	0.875	0.894	9.756
T1038	FM/TBM	0.868	0.873	0.831	0.851	0.876	1.829
T1046s1	FM/TBM	0.975	0.968	0.944	0.963	0.975	27.439
T1047s2	FM/TBM	0.669	0.720	0.692	0.706	0.724	3.658
T1052	FM/TBM	0.582	0.561	0.548	0.555	0.563	4.878
T1053	FM/TBM	0.893	0.937	0.9	0.916	0.938	1.829
T1055	FM/TBM	0.864	0.873	0.813	0.836	0.870	0
T1058	FM/TBM	0.858	0.836	0.8	0.820	0.839	5.487
T1061	FM/TBM	0.615	0.633	0.620	0.629	0.638	15.853
T1065s2	FM/TBM	0.989	0.977	0.933	0.958	0.974	0
T1070	FM/TBM	0.410	0.415	0.400	0.407	0.416	3.048
T1082	FM/TBM	0.953	0.976	0.92	0.960	0.98	5.48
T1093	FM/TBM	0.677	0.509	0.506	0.526	0.539	97.575
T1094	FM/TBM	0.707	0.673	0.667	0.680	0.693	92.682
T1024	TBM	0.600	0.593	0.571	0.582	0.593	0.609
T1026	TBM	0.934	0.885	0.811	0.834	0.883	0
T1030	TBM	0.628	0.636	0.609	0.622	0.637	1.2195
T1032	TBM	0.686	0.682	0.663	0.676	0.685	14.634
T1034	TBM	0.937	0.943	0.921	0.939	0.950	24.390
T1045s2	TBM	0.917	0.917	0.875	0.899	0.921	3.0487
T1046s2	TBM	0.964	0.957	0.934	0.951	0.966	21.951
T1050	TBM	0.860	0.799	0.774	0.786	0.801	3.658

T1054	TBM	0.921	0.898	0.867	0.883	0.898	3.658
T1056	TBM	0.952	0.849	0.825	0.835	0.849	1.829
T1060s2	TBM	0.827	0.774	0.753	0.766	0.777	9.756
T1060s3	TBM	0.951	0.920	0.900	0.925	0.943	78.048
T1065s1	TBM	0.958	0.960	0.924	0.945	0.958	0
T1067	TBM	0.893	0.895	0.855	0.879	0.896	3.048
T1068	TBM	0.962	0.967	0.946	0.958	0.969	2.439
T1073	TBM	0.839	0.826	0.783	0.808	0.826	2.439
T1076	TBM	0.990	0.986	0.950	0.963	0.986	3.048
T1078	TBM	0.959	0.967	0.949	0.963	0.970	28.048
T1079	TBM	0.916	0.913	0.880	0.896	0.913	1.219
T1083	TBM	0.856	0.877	0.847	0.864	0.883	7.317
T1084	TBM	0.912	0.915	0.887	0.905	0.926	17.682
T1087	TBM	0.967	0.825	0.79	0.813	0.828	5.487
T1089	TBM	0.970	0.974	0.952	0.963	0.976	3.655
T1092	TBM	0.738	0.495	0.49	0.505	0.512	98.780
T1095	TBM	0.704	0.7106	0.713	0.726	0.736	100
T1099	TBM	0.751	0.695	0.668	0.684	0.700	4.268
T1100	TBM	0.792	0.797	0.767	0.784	0.802	6.097
T1101	TBM	0.870	0.824	0.791	0.809	0.827	7.926
The Cumulative scores		46.861	45.0656	43.425	44.4068	45.325	

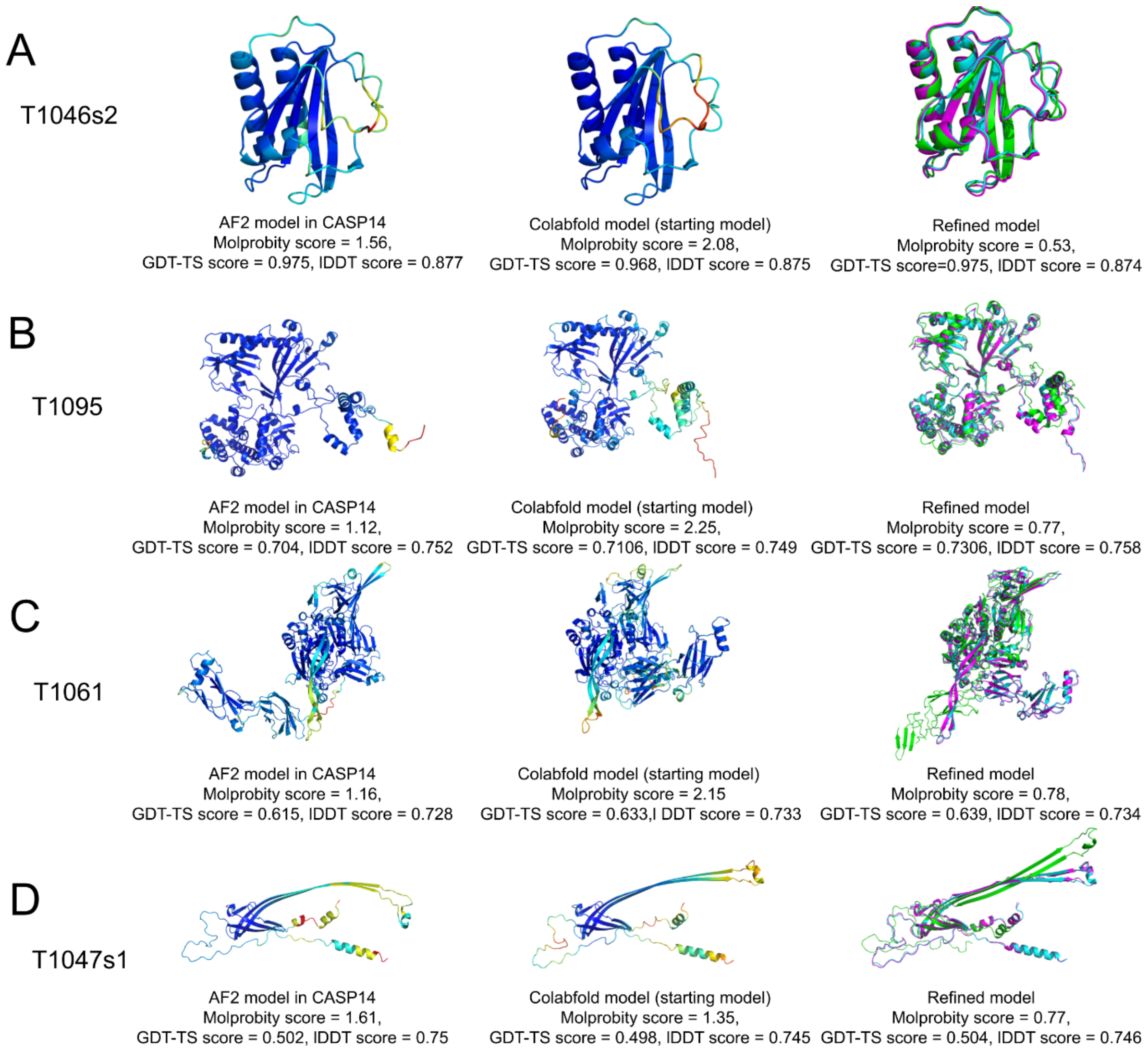

Supplementary Figure 1. The refinement of four example CASP14 targets using the ReFOLD4 protocol. The images in the left columns show the top models submitted by the AF2 group (427) in CASP14 and were coloured by the plDDT. The middle columns show the top models generated by LocalColabFold coloured by the plDDT, and these models were used as initial structures by ReFOLD4. The right columns show the superposition of the top 3D models generated by LocalColabFold (cyan), the best 3D models generated

by ReFOLD4 (magenta) and native structures (green). (A) CASP14 regular TBM T1046s2: the AF2 group model has a Molprobity score of 1.56, a GDT-TS score of 0.975, and a lDDT score of 0.877, the LocalColabfold model was refined using ReFOLD4, with a Molprobity improvement from 2.08 to 0.53, and a GDT-TS improvement from 0.968 to 0.975. (B) CASP14 regular TBM T1095: the AF2 group model has a Molprobity score of 1.12, a GDT-TS score of 0.704 and a lDDT score of 0.752, the LocalColabfold model was refined using ReFOLD4, with a Molprobity improvement from 2.25 to 0.77, and a GDT-TS improvement from 0.7106 to 0.7306. (C) CASP14 regular FM/TBM T1061: the AF2 group model has a Molprobity score of 1.16, a GDT-TS score of 0.615, and a lDDT score of 0.728, the LocalColabfold model was refined using ReFOLD4, with a Molprobity improvement from 2.15 to 0.78, and a GDT-TS improvement from 0.633 to 0.639. (D) CASP14 regular FM T1047s1: the AF2 group model has a Molprobity score of 1.61, a GDT-TS score of 0.502, and a lDDT score of 0.75, the LocalColabfold model was refined using ReFOLD4, with a Molprobity improvement from 1.35 to 0.77, and a GDT-TS improvement from 0.498 to 0.504. Images were rendered using PyMOL.

Supplementary Table 5. Performance summary for ReFOLD4 on the CASP14 targets according to lDDTscore. The starting 3D models generated by LocalColabFold were further refined by ReFOLD4

CASP Target ID	Prediction method	Score of model submitted by AF2 in CASP14	The starting model generated by ColabFold	Minimum score of refined models	Mean score of refined models	Maximum score of refined models	Percentage of improved models
T1027	FM	0.433	0.464	0.433	0.441	0.464	0.609
T1029	FM	0.472	0.472	0.456	0.461	0.470	0
T1031	FM	0.714	0.696	0.665	0.681	0.704	4.268
T1033	FM	0.818	0.757	0.709	0.722	0.749	0
T1037	FM	0.792	0.703	0.670	0.678	0.704	3.0487
T1039	FM	0.717	0.734	0.689	0.703	0.728	0
T1040	FM	0.740	0.635	0.605	0.616	0.633	0
T1041	FM	0.828	0.834	0.78	0.798	0.834	0
T1042	FM	0.831	0.673	0.635	0.645	0.673	2.439
T1043	FM	0.75	0.749	0.702	0.723	0.743	0
T1047s1	FM	0.75	0.745	0.712	0.722	0.746	3.0487
T1049	FM	0.848	0.868	0.792	0.810	0.86	0
T1064	FM	0.746	0.515	0.461	0.478	0.508	0
T1074	FM	0.836	0.846	0.761	0.776	0.836	0
T1090	FM	0.827	0.830	0.782	0.793	0.828	0

T1035	FM/TBM	0.868	0.824	0.757	0.781	0.813	0
T1038	FM/TBM	0.832	0.830	0.777	0.789	0.825	0
T1046s1	FM/TBM	0.894	0.882	0.816	0.837	0.88	0
T1047s2	FM/TBM	0.775	0.788	0.747	0.758	0.787	0
T1052	FM/TBM	0.864	0.853	0.800	0.810	0.854	0
T1053	FM/TBM	0.860	0.853	0.806	0.815	0.851	0
T1055	FM/TBM	0.737	0.746	0.649	0.680	0.730	0
T1058	FM/TBM	0.826	0.813	0.765	0.775	0.811	0
T1061	FM/TBM	0.728	0.733	0.710	0.716	0.734	3.048
T1065s2	FM/TBM	0.909	0.913	0.818	0.849	0.905	0
T1070	FM/TBM	0.730	0.734	0.691	0.701	0.735	2.439
T1082	FM/TBM	0.866	0.870	0.793	0.820	0.857	0
T1093	FM/TBM	0.749	0.737	0.730	0.735	0.741	9.756
T1094	FM/TBM	0.754	0.745	0.732	0.738	0.743	0
T1024	TBM	0.781	0.772	0.733	0.743	0.772	2.439
T1026	TBM	0.808	0.723	0.658	0.672	0.721	0
T1030	TBM	0.850	0.876	0.812	0.829	0.873	0
T1032	TBM	0.72	0.723	0.676	0.688	0.722	0
T1034	TBM	0.853	0.864	0.813	0.826	0.863	0
T1045s2	TBM	0.852	0.853	0.792	0.810	0.851	0
T1046s2	TBM	0.877	0.875	0.815	0.831	0.874	0
T1050	TBM	0.871	0.868	0.819	0.828	0.869	3.658
T1054	TBM	0.869	0.857	0.790	0.812	0.856	0
T1056	TBM	0.904	0.812	0.740	0.757	0.811	0
T1060s2	TBM	0.897	0.897	0.833	0.856	0.892	0
T1060s3	TBM	0.815	0.804	0.780	0.794	0.806	3.048
T1065s1	TBM	0.897	0.897	0.833	0.856	0.892	0

T1067	TBM	0.869	0.866	0.802	0.820	0.865	0
T1068	TBM	0.905	0.908	0.834	0.852	0.899	0
T1073	TBM	0.771	0.768	0.680	0.707	0.756	0
T1076	TBM	0.943	0.937	0.864	0.875	0.935	0
T1078	TBM	0.922	0.941	0.857	0.880	0.936	0
T1079	TBM	0.909	0.915	0.861	0.870	0.915	1.219
T1083	TBM	0.782	0.817	0.753	0.774	0.812	0
T1084	TBM	0.892	0.864	0.804	0.827	0.8613	0
T1087	TBM	0.9	0.734	0.680	0.696	0.73	0
T1089	TBM	0.917	0.924	0.864	0.877	0.9217	0
T1092	TBM	0.766	0.775	0.773	0.777	0.7844	89.634146
T1095	TBM	0.752	0.749	0.748	0.752	0.758	98.17073
T1099	TBM	0.779	0.749	0.710	0.723	0.7501	3.0487804
T1100	TBM	0.865	0.852	0.805	0.8184	0.8524	1.219512
T1101	TBM	0.859	0.856	0.787	0.801	0.853	0
The Cumulative scores		46.342	45.347	42.387	43.227	45.1978	

Supplementary Table 6. Calculated p-values, based on lDDT scores (A) and TM-scores (B), for recycled models for CASP14 AF2 and non-AF models for monomeric targets.

A								
Models	Recycle type	Baseline to 1 recycle	1 recycle to 3 recycles	Baseline to 3 recycles	3 recycles to 6 recycles	Baseline to 6 recycles	6 recycles to 12 recycles	Baseline to 12 recycles.
AF2	MSA	1.87e-01	7.56e-01	**5.66e-03**	4.36e-02	**7.72e-03**	3.51e-01	**1.30e-02**
	SS	**1.12e-02**	9.54e-01	**1.86e-02**	1.24e-01	5.90e-02	6.37e-01	**3.86e-02**
non-AF	MSA	**1.23e-09**	**1.21e-08**	**7.10e-15**	**1.56e-02**	**7.10e-15**	4.73e-01	**1.23e-09**
	SS	**1.70e-09**	**4.91e-05**	**1.50e-09**	1.75e-01	**1.50e-09**	5.87e-01	**1.40e-09**
B								
Models	Recycle type	Baseline to 1 recycle	1 recycle to 3 recycles	Baseline to 3 recycles	3 recycles to 6 recycles	Baseline to 6 recycles	6 recycles to 12 recycles	Baseline to 12 recycles.
AF2	MSA	6.79e-01	8.01e-01	7.96e-01	1.06e-01	9.58e-01	3.63e-01	7.76e-01
	SS	7.17e-01	9.09e-01	8.60e-01	**3.37e-02**	8.97e-01	7.82e-01	6.98e-01
non-AF	MSA	**1.42e-14**	**6.31e-05**	**4.36e-12**	8.98e-01	**8.05e-09**	2.40e-01	**2.40e-12**
	SS	**5.13e-07**	**7.61e-05**	**3.35e-07**	**3.30e-02**	**1.98e-07**	6.60e-01	**1.62e-07**

*SS=Single sequence. Ho: Recycling as specified per column produces models that are no different in quality to those input as baseline templates or to the previous recycle number. H1: Recycling as specified per column produces higher quality models to those input at baseline or to the previous recycle number. P-values ≤0.05 indicate significant differences (in bold) were calculated by a 1-tailed Wilcoxon signed-rank test using lDDT score (A) and TM-scores (B) for 16 AlphaFold2 CASP14 top-ranked models (AF2) and 47 non-AlphaFold models (Non-AF) from the same CASP14 targets
.

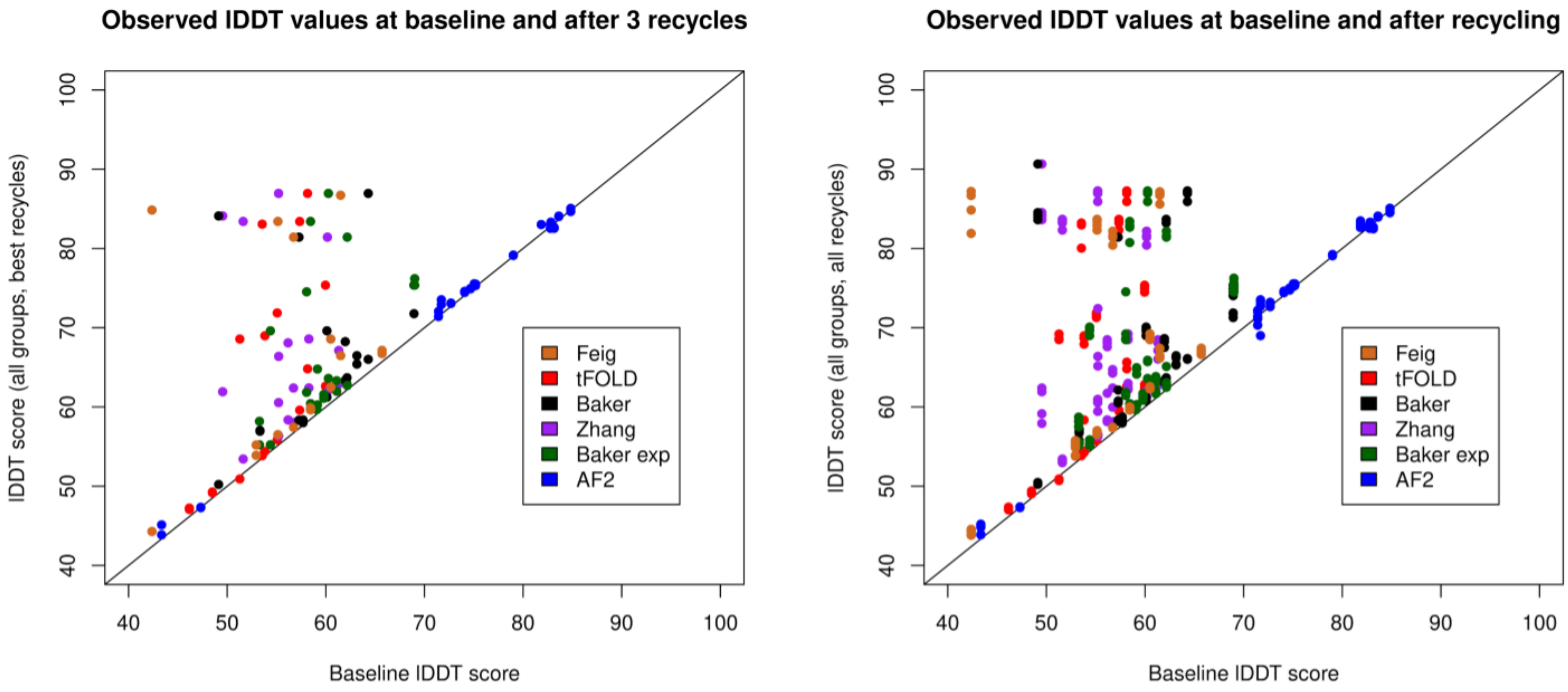

Supplementary Figure 2. Scatter plots showing observed lDDT scores for all models between baseline and recycle 3 (left) and baseline and all recycles (right) for both recycle models, coloured by group.

Supplementary Table 7. A comparison matrix of single-sequence versus MSA recycling by comparing mean lDDT scores and scale parameters across 1, 3, 6 and 12 recycles for CASP14 AF2 and non-AF2 models.

	p-values (single sequence vs. MSA)			
**AF2 models**	Recycle 1	Recycle 3	Recycle 6	Recycle 12
Wilcox signed rank	0.097	0.052	0.111	0.129
Ansari test	0.397	0.500	0.425	0.544
**non-AF2 models**	p-values (single sequence vs. MSA)			
Wilcox signed rank	**1.42×10^{-14}**	**5.34×10^{-9}**	**2.94×10^{-12}**	**7.80×10^{-9}**
Ansari test	**0.014**	**0.015**	**0.019**	**0.012**

Ho: Recycling using the single sequence setting produces models that are equal in quality to those produced using the MSA setting for equivalent recycle numbers. H1: Recycling using the single sequence setting produces models that are lower in quality to those produced using the MSA setting for equivalent recycle numbers. P-values ≤0.05 indicate significant differences. The 1-tailed Wilcoxon signed-rank sum test and 1-tailed Ansari-Bradley test were used to calculate p-values from lDDT scores across 16 AlphaFold CASP14 top-ranked models (upper two rows) and 47 non-AlphaFold CASP14 top-ranked models (lower two rows).

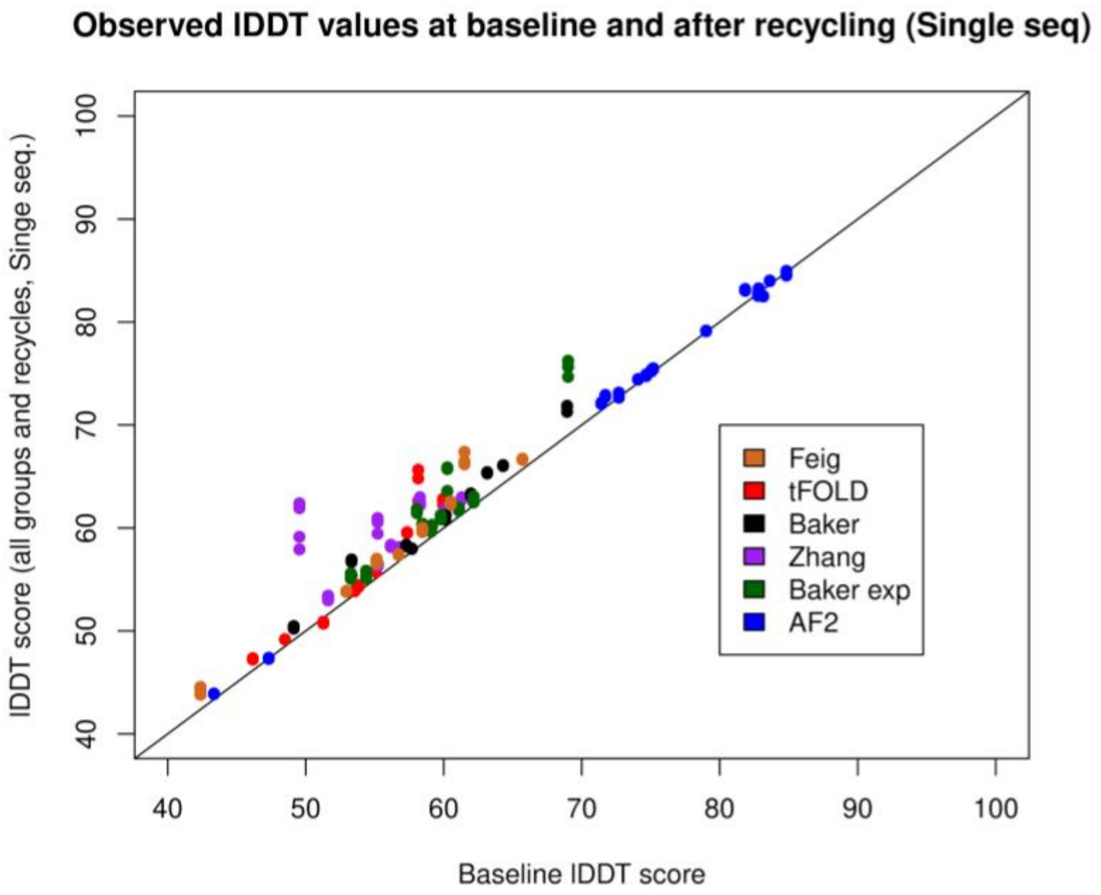

Supplementary Figure 3. Scatter plot to show comparisons in observed lDDT scores between baseline and all recycles for all models (AF2 and non-AF2).

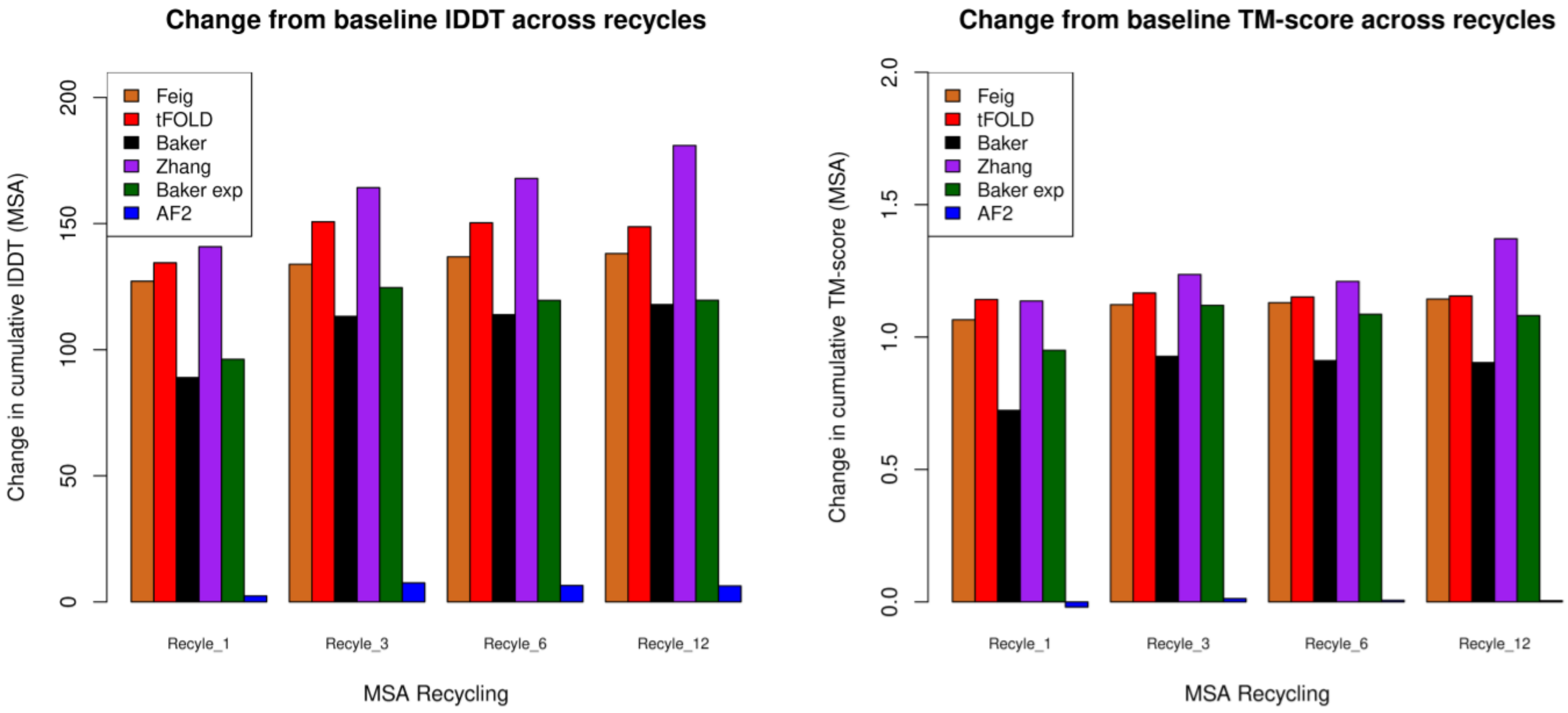

Supplementary Figure 4. Bar charts showing the cumulative increase in observed lDDT scores (A) and TM-scores (B) from the baseline models to the models produced using different numbers of recycles. Bars are coloured by group.

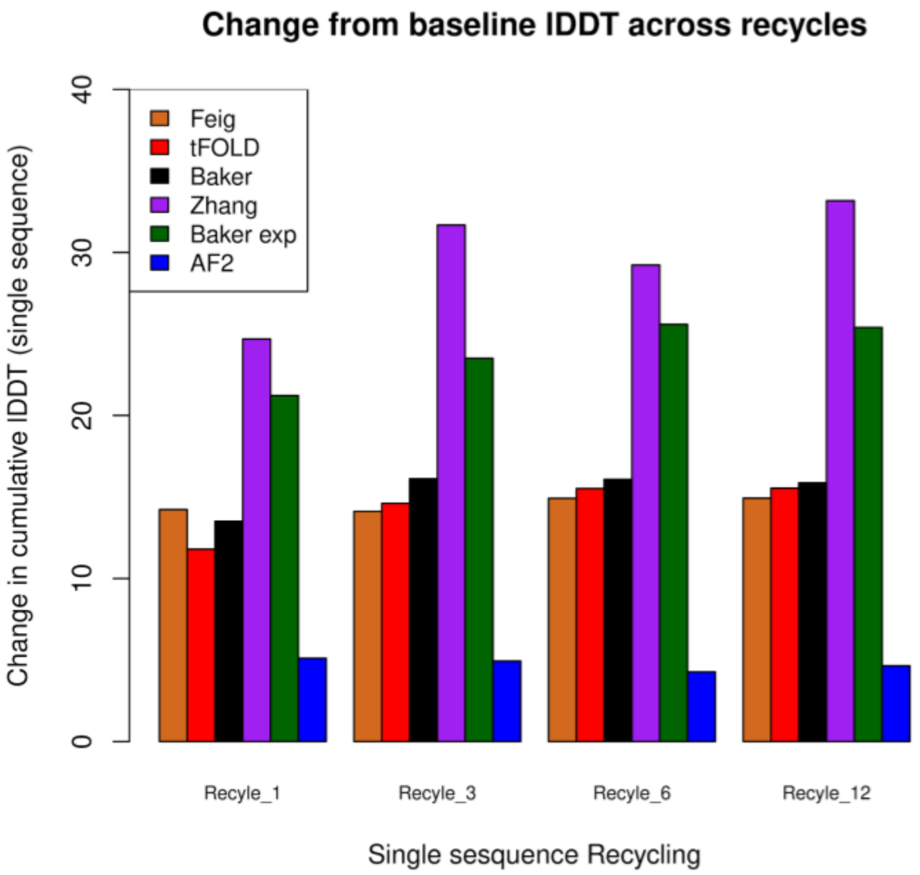

Supplementary Figure 5. Graphs to show average change in IDDT scores from baseline over all recycles for the individual CASP14 and AF2 models- single sequence modelling.

Supplementary Table 8. Calculated p-values based on oligo-lDDT scores (A), TM scores (B), QS-scores (C), for baseline and recycled models for both CASP14 non-AF2 models and the AF2-Multimer models for multimeric targets.

A								
Models	Recycle type	Baseline to 1 recycle	1 recycle to 3 recycles	Baseline to 3 recycles	3 recycles to 6 recycles	Baseline to 6 recycles	6 recycles to 12 recycles	Baseline to 12 recycles
AF2M	MSA	1.106e-1	5.203e-1	1.795e-1	7.679e-2	**4.157e-2**	1.106e-1	**5.146e-2**
	SS	9.966e-1	**4.157e-2**	9.369e-1	6.177e-2	9.369e-1	9.736e-1	9.369e-1
non-AF2M	MSA	**3.748e-3**	**4.267e-05**	**1.398e-05**	**6.915e-3**	**1.02e-06**	9.565e-1	**4.935e-07**
	SS	8.492e-1	**1.475e-2**	5.116e-1	1.611e-1	4.197e-1	**1.013e-2**	3.285e-1
B								
AF2M	MSA	**3.327e-2**	2.704e-1	6.314e-2	6.314e-2	**2.075e-2**	8.205e-1	1.54e-1
	SS	**5.146e-2**	1.795e-1	**2.075e-2**	6.201e-1	**1.247e-2**	6.202e-1	**2.075e-2**
non-AF2M	MSA	**2.066e-0**9	2.715e-1	**1.453e-09**	9.428e-1	**2.926e-09**	9.858e-1	**6.889e-09**
	SS	**3.338e-3**	**3.97e-3**	**5.516e-4**	7.458e-1	**1.367e-4**	3.75e-1	**2.946e-4**
C								
AF2M	MSA	4.161e-1	5.724e-1	1.976e-1	4.27e-1	5e-1	5e-1	5e-1
	SS	7.992e-1	**5.017e-2**	5e-1	1.855e-1	3.422e-1	8.618e-1	3.375e-1
non-AF2M	MSA	**1.577e-07**	2.268e-1	**2.578e-07**	1.575e-1	**1.09e-07**	2.326e-1	**6.799e-08**
	SS	**3.491e-2**	**1.118e-2**	**4.175e-3**	3.083e-1	**2.548e-3**	2.406e-1	**4.089e-3**

*SS=Single sequence. Ho: Recycling by the number specified per column produces models that are equal or lower in quality than those input as baseline templates or by the previous recycle number. H1: Recycling by the number specified per column produces higher quality models than those input at baseline or by the previous recycle number. P-values ≤0.05 indicate significant differences (in bold). The 1-tailed Wilcoxon signed-rank sum test P-values were calculated using lDDT scores (A), TM-scores (B), QS-scores (C) across 10 AlphaFold models (10 AlphaFold generated by MSA for the MSA method and 10 AlphaFold generated by Single sequence for the Single sequence method) and 50 non-AlphaFold models from different CASP14 targets.

A)

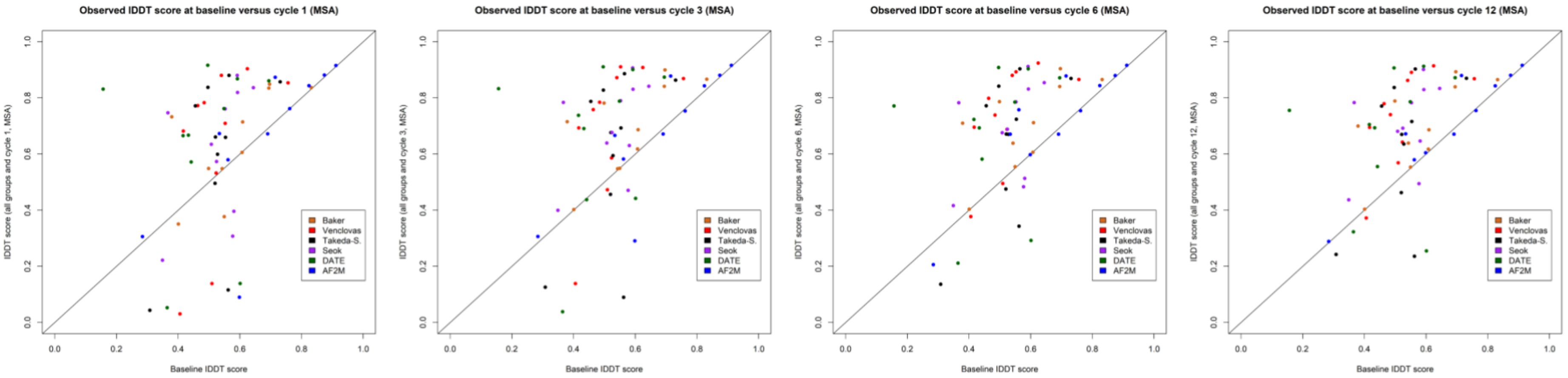
Observed lDDT score at baseline versus cycle 1 (MSA)
Observed lDDT score at baseline versus cycle 3 (MSA)
Observed lDDT score at baseline versus cycle 6 (MSA)
Observed lDDT score at baseline versus cycle 12 (MSA)
lDDT score (all groups and cycle 1, MSA)
lDDT score (all groups and cycle 3, MSA)
lDDT score (all groups and cycle 6, MSA)
lDDT score (all groups and cycle 12, MSA)
Baseline lDDT score
Baker
Venclovas
Takeda-S.
Seok
DATE
AF2M

B)

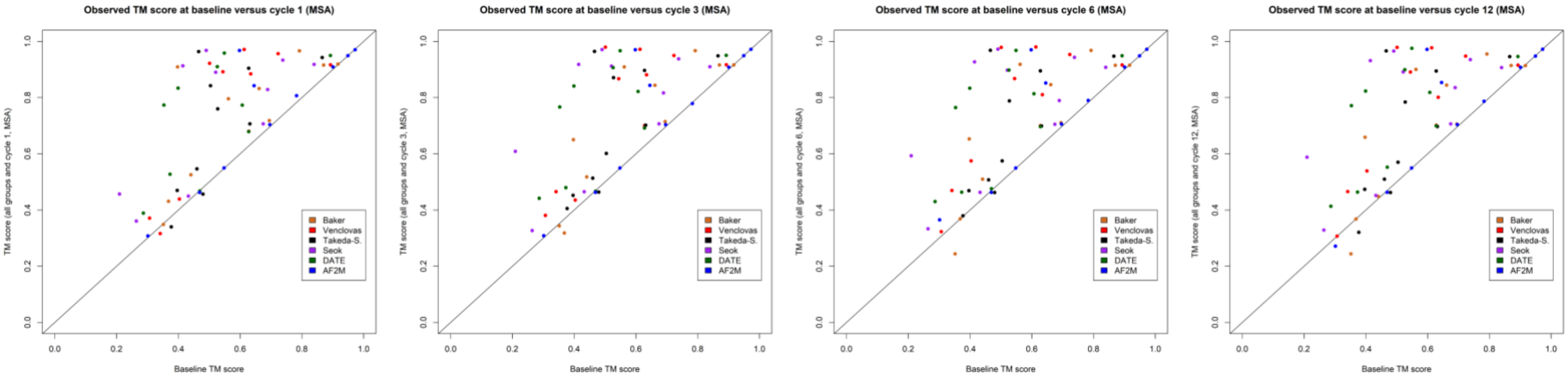
Observed TM score at baseline versus cycle 1 (MSA)
Observed TM score at baseline versus cycle 3 (MSA)
Observed TM score at baseline versus cycle 6 (MSA)
Observed TM score at baseline versus cycle 12 (MSA)
TM score (all groups and cycle 1, MSA)
TM score (all groups and cycle 3, MSA)
TM score (all groups and cycle 6, MSA)
TM score (all groups and cycle 12, MSA)
Baseline TM score
Baker
Venclovas
Takeda-S.
Seok
DATE
AF2M

C)

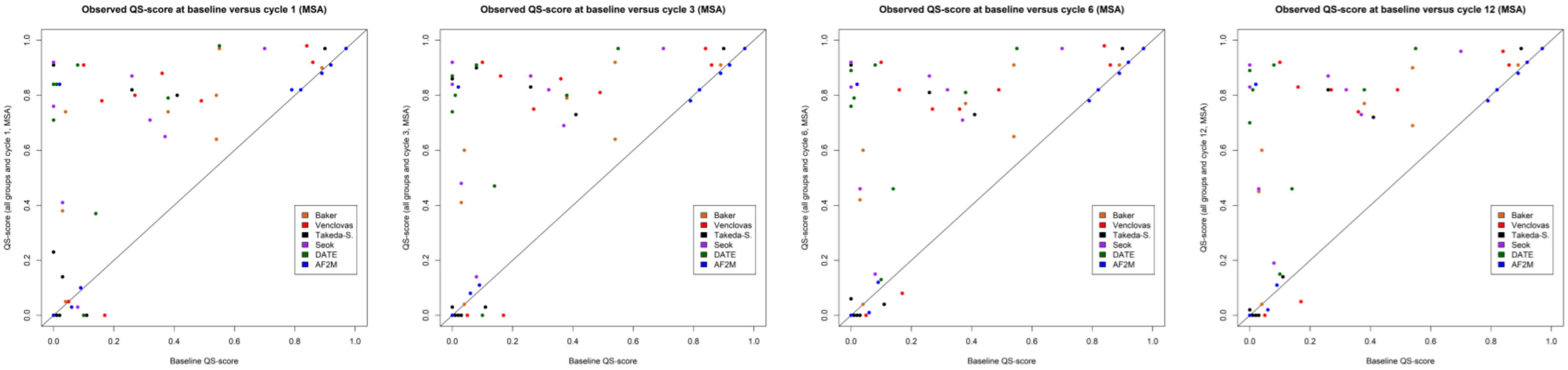

Supplementary Figure 6. Graphs to show comparisons in observed lDDT scores (A), TM scores (B), QS-scores (C) between baseline and each recycle separately for all models (AF2M and non-AF2) in the MSA methods.

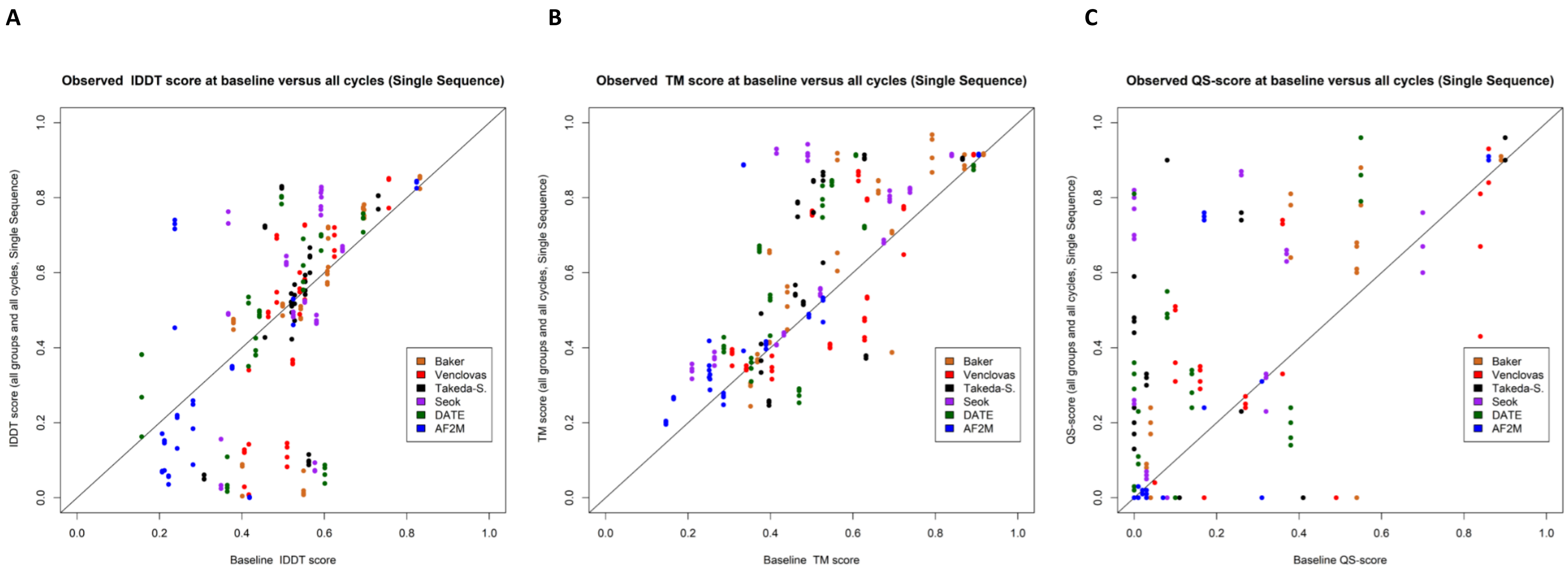

Supplementary Figure 7. Graphs to show comparisons in observed lDDT scores (A), TM scores (B), QS-scores (C) between baseline and all recycle for all models (AF2M and non-AF2).

A)

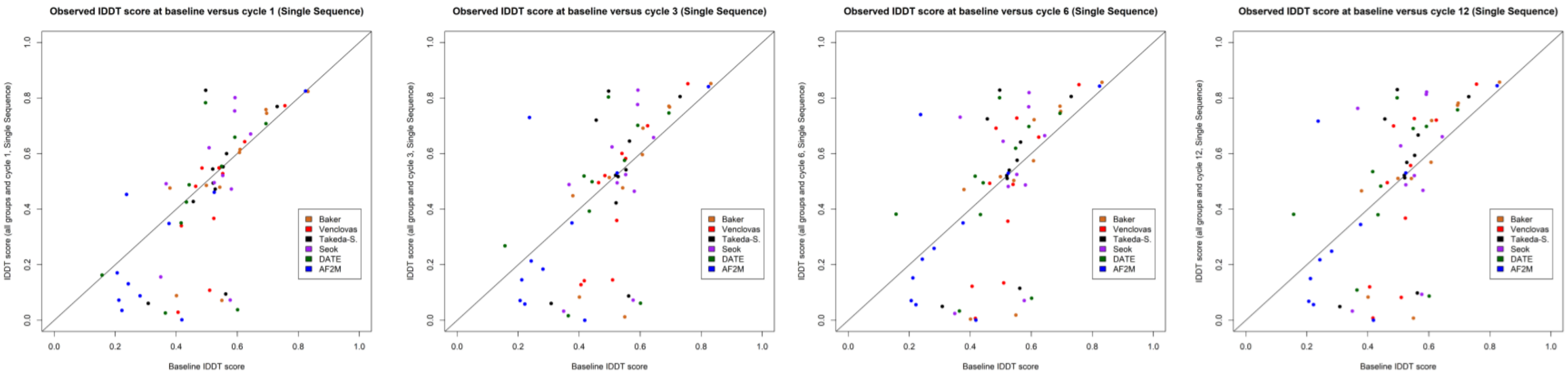
Observed IDDT score at baseline versus cycle 1 (Single Sequence)
Observed IDDT score at baseline versus cycle 3 (Single Sequence)
Observed IDDT score at baseline versus cycle 6 (Single Sequence)
Observed IDDT score at baseline versus cycle 12 (Single Sequence)
IDDT score (all groups and cycle 1, Single Sequence)
IDDT score (all groups and cycle 3, Single Sequence)
IDDT score (all groups and cycle 6, Single Sequence)
IDDT score (all groups and cycle 12, Single Sequence)
Baseline IDDT score
Baker
Venclovas
Takeda-S.
Seok
DATE
AF2M

B)

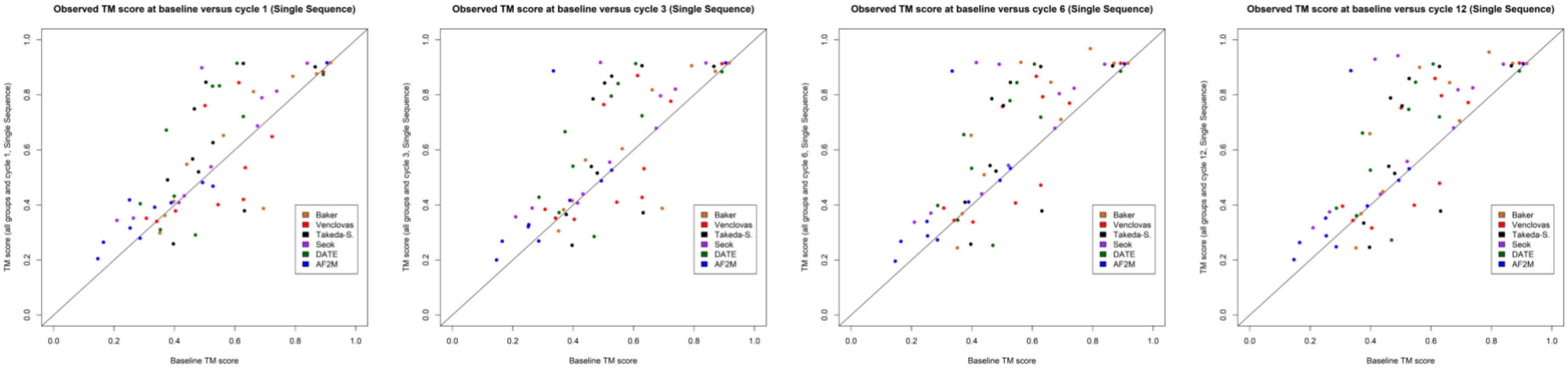
Observed TM score at baseline versus cycle 1 (Single Sequence)
Observed TM score at baseline versus cycle 3 (Single Sequence)
Observed TM score at baseline versus cycle 6 (Single Sequence)
Observed TM score at baseline versus cycle 12 (Single Sequence)
TM score (all groups and cycle 1, Single Sequence)
TM score (all groups and cycle 3, Single Sequence)
TM score (all groups and cycle 6, Single Sequence)
TM score (all groups and cycle 12, Single Sequence)
Baseline TM score
Baker
Venclovas
Takeda-S.
Seok
DATE
AF2M

C)

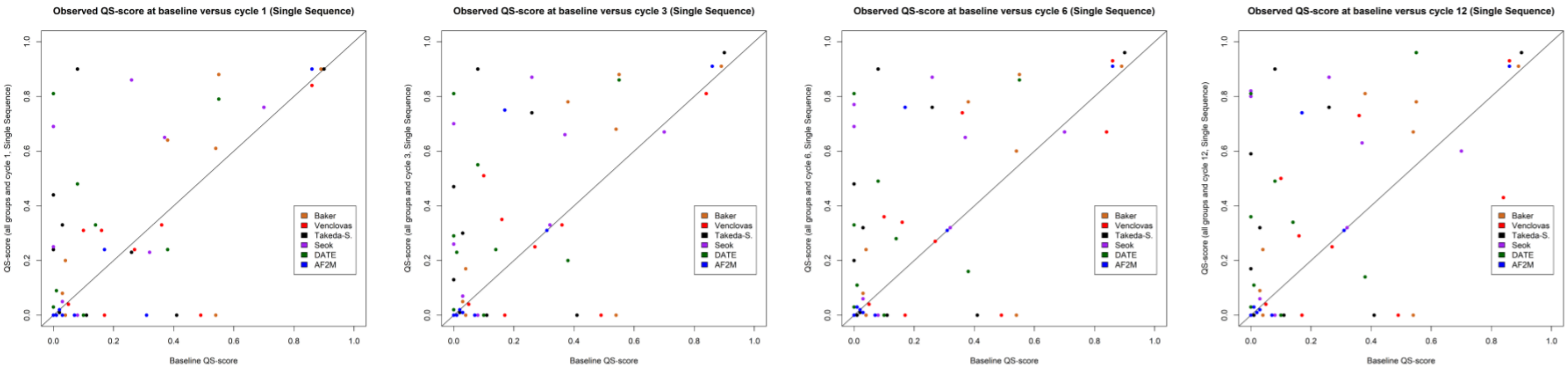

Supplementary Figure 8. Graphs to show comparisons observed lDDT scores (A), TM scores (B), QS-scores (C) between baseline and each recycle separately, for all models (AF2M and non-AF2) in the Single Seq.

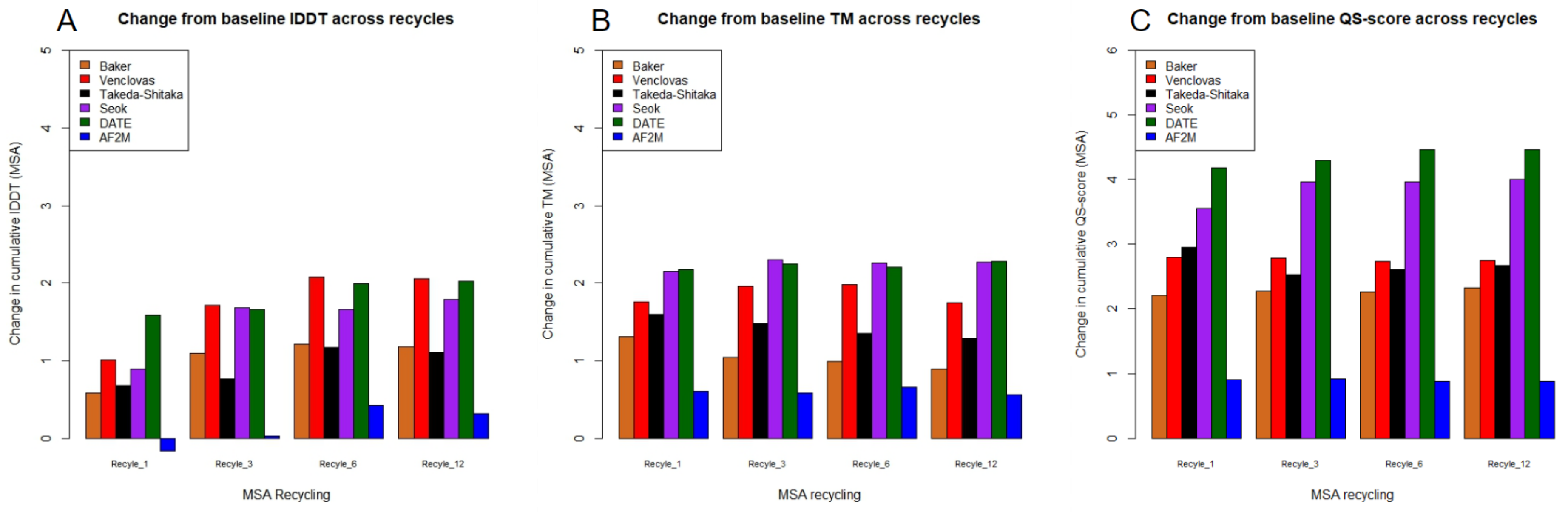

Supplementary Figure 9. Bar charts showing the cumulative increase in observed oligo-lDDT scores (A), TM-scores (B) and QS score (C) from the baseline multimer models to the models produced using different numbers of recycles. Bars are coloured by group.

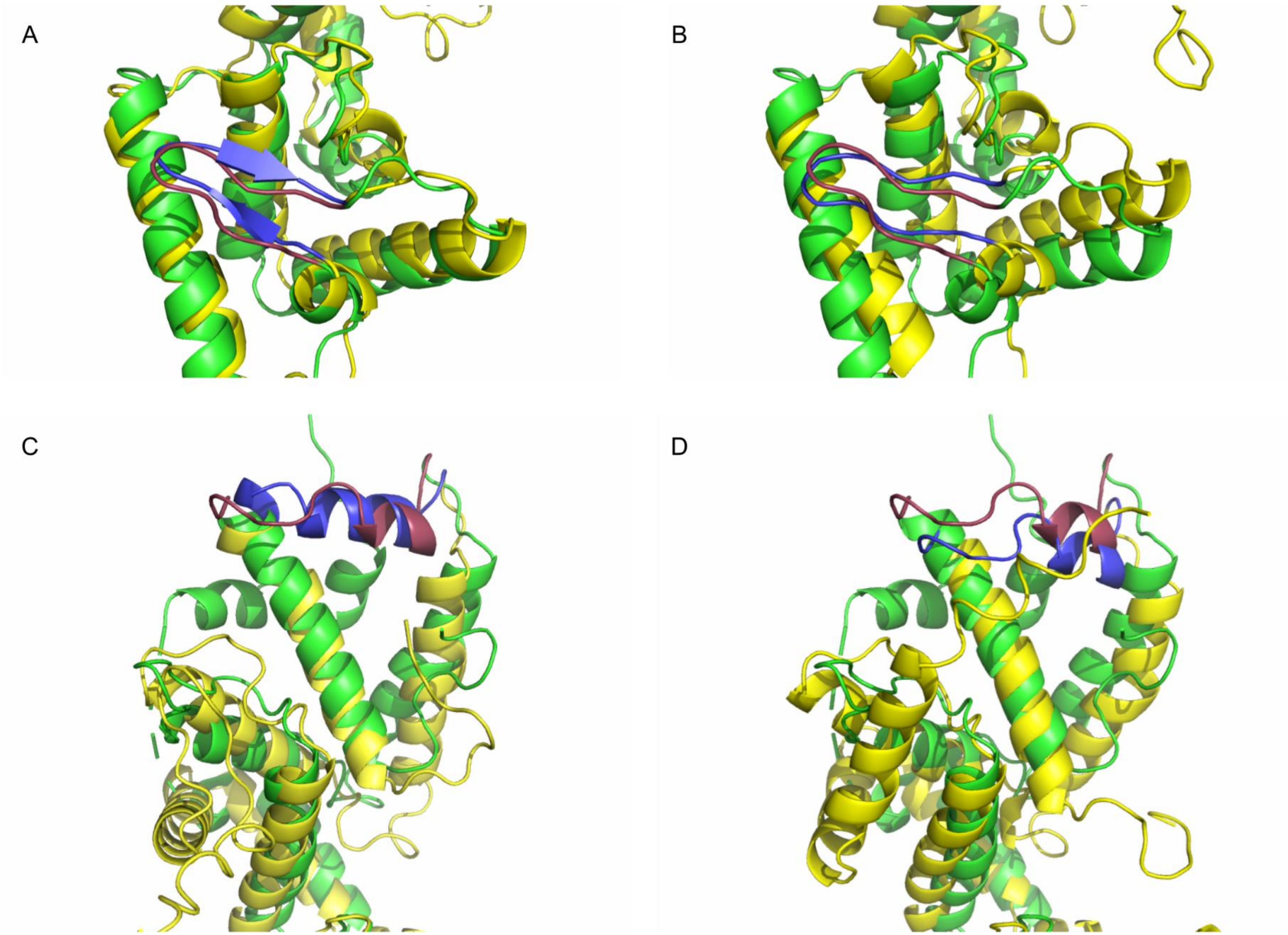
A
B
C
D

Supplementary Figure 10. Images of Zhang (group 129) T1042 MSA models which make up the three outliers seen in Figure 1B. These all increased in lDDT score while simultaneously decreasing in TM-score (the 3-Recycle model (0.660), 6-Recycle model (0.666) and 12-Recycle model (0.684), all down from 0.726 at baseline). Here we compare the 1-Recycle model, which showed a slight increase from baseline in TM-score with the 3-Recycle model which showed the largest decrease in TM-score. The upper images (panels A and B) show the differences in residues 160 to 170 between the 1 and 3-recycle models. A) The superposition of the native structure (green, with residues 160-170 in red) with the 1-recycled model (yellow, residues 160-170 in blue) showing an incorrectly placed beta strands in the 1-recycle model. B) The superposition of the same section between the native structure (green and red) with the 3-recycle model (yellow and blue) showing that the beta strands are now correctly replaced with a loop (the lDDT scores for this section increased by a total of 1.163). However, it is also evident that some changes to the surrounding two helices have occurred simultaneously, possibly to accommodate this change, illustrating how it is possible for the 3-recycle model to score higher by lDDT but lower by TM-score.

The lower images (C and D) show the same T1042 models, this time illustrating the differences in residues 31 to 46 between the 1 and 3-recycle models. C) The superposition of the native structure (green, with residues 31-46 in red) with the 1-recycle model (yellow, residues 31-46 in blue) showing a small helix and loop region in the native structure but a much larger helix for the 1-recycle model. D) The superposition of the same section between the native structure (green and red) with the 3-recycle model (yellow and blue) showing a much closer match in the helix and loop region between the two structures (the lDDT scores for this section increased by a total of 3.235).

Supplementary Table 9. The four tables present molprobity score(lower Molprobity scores are better) of CASP14 target, with cycle 1-3-6-12 and baseline as starting model A) Molprobity scores of monomeric targets received via MSA B) Molprobity scores of monomeric targets received via Single sequence C) Molprobity scores of multimeric targets received via MSA D) Molprobity scores of multimeric targets received via Single sequence. Molprobity score was obtained via http://molprobity.biochem.duke.edu/. Monomeric targets were sequenced based on group, whereas in multimeric targets, sequencing was performed based on targets.

A)

**Molprobity of monomeric targets (MSA)**						
TARGET	Group	Baseline	R_1	R_3	R_6	R_12
T1031	Baker	0.71	2.99	2.14	3.21	2.34
T1033	Baker	0.84	2.93	2.54	2.51	2.44
T1037	Baker	1.07	2.25	2.22	2.23	2.28
T1039	Baker	0.74	2.68	2.26	2.3	2.34
T1041	Baker	0.75	1.91	2.41	2.05	1.95
T1042	Baker	1.22	2.35	2.39	2.53	2.5
T1043	Baker	1.29	2.61	2.21	2.38	2.26
T1049	Baker	0.95	2.4	2.26	2.24	2.08
T1074	Baker	0.67	3.48	3.01	3.06	3.25
T1090	Baker	0.99	2.29	2.22	1.65	1.68
T1027	Baker_exp	1.01	2.75	2.91	2.78	2.77
T1031	Baker_exp	1.38	2.99	3.12	3.21	2.68
T1033	Baker_exp	1.06	3.04	2.54	2.51	2.44
T1037	Baker_exp	1.25	2.41	2.41	2.37	2.35
T1039	Baker_exp	0.69	2.68	2.26	2.3	2.34
T1041	Baker_exp	1	2.52	2.41	2.46	2.1
T1042	Baker_exp	1.15	2.93	2.52	2.51	2.5
T1043	Baker_exp	1.18	2.59	2.83	3.03	2.97
T1049	Baker_exp	1.14	2.4	2.27	2.24	2.08

T1090	Baker_exp	1.25	2.41	1.82	1.78	1.85
T1096	Baker_exp	1.18	2.03	2	1.88	1.97
T1031	Feig	0.54	2.99	2.86	3.21	2.68
T1037	Feig	1.01	2.44	2.46	2.4	2.5
T1041	Feig	0.89	2.41	2.41	2.46	2.1
T1042	Feig	0.85	2.39	2.61	2.35	2.33
T1049	Feig	0.99	2.33	2.17	2.11	2.08
T1074	Feig	0.91	3.37	3.17	3.21	3.16
T1090	Feig	1.01	2.18	1.82	1.65	2.12
T1096	Feig	0.84	2.29	2.28	2.22	2.27
T1029	tFOLD	1.89	1.78	1.99	1.93	1.93
T1031	tFOLD	2.25	2.99	2.86	3.21	2.69
T1033	tFOLD	1.94	3.04	2.54	2.51	2.44
T1037	tFOLD	2.53	2.85	2.79	2.65	2.67
T1041	tFOLD	1.71	2.36	2.35	2.36	2.38
T1042	tFOLD	1.03	2.73	3.01	2.91	2.79
T1043	tFOLD	1.12	1.79	2.05	2.12	1.71
T1049	tFOLD	1.79	2.4	2.27	2.24	2.08
T1090	tFOLD	1.31	2.18	1.82	1.65	1.68
T1031	Zhang	1.05	2.99	2.86	3.21	2.68
T1037	Zhang	1.16	2.58	2.79	2.52	2.53
T1040	Zhang	1.48	3.44	3.13	3.12	2.93
T1041	Zhang	1.08	2.41	2.41	2.46	2.1
T1042	Zhang	1.45	2.89	2.94	3.09	2.78
T1049	Zhang	1.47	2.4	2.27	2.24	2.08

T1074	Zhang	1	3.48	3.01	3.06	3.25
T1090	Zhang	1.63	2.18	1.82	1.65	1.68
T1096	Zhang	1.18	2.48	2.56	2.59	2.68
T1027	AF2	2.57	2.93	3	2.97	2.9
T1029	AF2	0.79	1.33	1.36	1.33	1.36
T1031	AF2	1.62	2.42	2.62	2.49	2.82
T1033	AF2	1.51	1.77	2.04	2.05	2.04
T1037	AF2	0.9	1.64	1.74	1.75	1.73
T1039	AF2	1.84	2.68	2.31	2.38	2.35
T1040	AF2	0.5	1.25	1.31	1.42	1.37
T1041	AF2	1.06	1.46	1.39	1.34	1.36
T1042	AF2	1.41	1.95	1.89	1.89	1.9
T1043	AF2	1.2	1.8	1.82	1.79	1.82
T1047s1	AF2	1.61	1.83	1.93	1.99	1.86
T1049	AF2	0.74	2.17	2.12	2.14	2.12
T1064	AF2	1.32	2.01	1.98	1.97	1.8
T1074	AF2	1.61	2.29	2.21	2.1	2.09
T1090	AF2	0.68	1.58	1.54	1.49	1.54
T1096	AF2	1.1	1.81	1.76	1.99	1.72
Cumulative score		1.207	2.427	2.333	2.340	2.258

B)

**Molprobity of monomeric targets (Single-Sequence)**						
TARGET	Group	Baseline	R_1	R_3	R_6	R_12
T1031	Baker	0.84	1.45	1.51	1.48	1.39

T1033	Baker	1.07	1.85	2.17	2.16	2.18
T1037	Baker	0.74	2.16	2.18	2.23	2.17
T1039	Baker	0.75	1.85	1.77	1.75	1.85
T1041	Baker	1.22	1.98	1.99	1.94	1.95
T1042	Baker	1.29	2.61	2.5	2.75	2.68
T1043	Baker	0.95	2.48	2.23	2.27	2.25
T1049	Baker	0.67	2.12	2.3	2.46	2.59
T1074	Baker	0.99	3.11	3.02	3.08	3
T1090	Baker	1.01	2.16	2.04	1.92	1.88
T1027	Baker_exp	0.71	3.52	3.62	3.3	2.73
T1031	Baker_exp	1.38	2.43	2.53	2.88	1.39
T1033	Baker_exp	1.06	2.72	2.72	2.75	3
T1037	Baker_exp	1.25	2.46	2.43	2.4	2.39
T1039	Baker_exp	0.69	2.03	1.99	1.93	1.91
T1041	Baker_exp	1	2.35	2.38	2.28	2.29
T1042	Baker_exp	1.15	2.4	2.28	2.17	2.27
T1043	Baker_exp	1.18	2.72	2.73	2.87	2.84
T1049	Baker_exp	1.14	2.62	2.57	3.24	3.23
T1090	Baker_exp	1.25	2.52	2.59	2.62	2.66
T1096	Baker_exp	1.18	2.35	2.16	2.11	2.08
T1031	Feig	0.54	2.03	2.05	2.18	2.2
T1037	Feig	1.01	2.4	2.39	2.39	2.38
T1041	Feig	0.89	2.31	2.12	2.17	2.18
T1042	Feig	0.85	2.71	2.4	2.46	2.46
T1049	Feig	0.99	2.56	2.59	3.05	3.03

T1074	Feig	0.91	2.87	3.12	3.28	3.24
T1090	Feig	1.01	2.6	2.62	2.63	2.63
T1096	Feig	0.84	2.04	2.19	2.14	2.14
T1029	tFOLD	1.89	2.17	2.16	2.23	2.24
T1031	tFOLD	2.25	2.32	2.55	2.56	2.5
T1033	tFOLD	1.94	2.28	2.56	2.92	2.73
T1037	tFOLD	2.53	2.76	2.85	2.73	2.73
T1041	tFOLD	1.71	1.77	1.75	1.86	1.88
T1042	tFOLD	1.03	2.08	2.13	2.17	2.26
T1043	tFOLD	1.12	1.95	2.02	2.12	2.1
T1049	tFOLD	1.79	2.63	3.23	3.27	3.14
T1090	tFOLD	1.31	2.81	2.8	2.8	2.6
T1031	Zheng	1.05	2.76	3.11	3.17	2.78
T1037	Zheng	1.16	2.43	2.51	2.51	2.5
T1040	Zheng	1.48	2.97	3.18	3.19	3.29
T1041	Zheng	1.08	2.27	2.34	2.34	2.38
T1042	Zheng	1.45	2.94	2.96	2.86	2.83
T1049	Zheng	1.47	2.85	3.2	3.07	3.17
T1074	Zheng	1	3.63	3.87	3.75	3.81
T1090	Zheng	1.63	3.14	2.94	2.87	3.11
T1096	Zheng	1.18	2.37	2.4	2.33	2.34
T1027	AF2	2.57	2.87	3.17	3.08	3.09
T1029	AF2	0.79	1.54	1.52	1.48	1.41
T1031	AF2	1.62	1.78	1.82	2.25	2.23
T1033	AF2	1.51	1.61	1.87	2	1.99

T1037	AF2	0.9	1.68	1.68	1.66	1.67
T1039	AF2	1.84	2.37	2.32	2.33	2.35
T1040	AF2	0.5	1.52	1.48	1.44	1.66
T1041	AF2	1.06	1.56	1.58	1.61	1.69
T1042	AF2	1.41	2.16	2.1	2.08	2
T1043	AF2	1.2	1.61	1.61	1.52	1.63
T1047s1	AF2	1.61	2.02	1.99	1.99	2.01
T1049	AF2	0.74	2.01	2.07	2.13	2.12
T1064	AF2	1.32	1.61	2.01	2	1.98
T1074	AF2	1.61	1.99	2.15	2.22	2.25
T1090	AF2	0.68	1.5	1.52	1.5	1.52
T1096	AF2	1.1	1.78	1.68	1.99	1.86
Cumulative score		1.207	2.303	2.354	2.395	2.362

C)

**Molprobity of multimeric targets (MSA)**						
TARGET	Group	Baseline	R_1	R_3	R_6	R_12
H1045	Baker	0.88	2.32	2.22	2.08	2.09
H1045	Venclovas	2.33	1.84	1.81	1.77	1.76
H1045	Takeda-Shitaka	2.85	2.07	1.93	1.9	1.83
H1045	Seok	1.5	2.18	1.87	1.82	1.82
H1045	DATE	2.52	1.92	1.86	1.84	1.86
H1045	AF2	3.84	2.11	2.15	2.18	2.09
H1065	Baker	0.9	1.79	1.7	1.62	1.64
H1065	Venclovas	0.76	1.64	1.69	1.49	1.45

H1065	Takeda-Shitaka	2.6	1.67	1.69	1.58	1.63
H1065	Seok	1.4	1.86	1.56	1.49	1.45
H1065	DATE	1.96	1.64	1.53	1.45	1.48
H1065	AF2	1.53	3.23	3.03	2.84	2.75
H1072	Baker	0.81	2.03	1.92	2.02	2.02
H1072	Venclovas	1.89	2.14	2.14	2.1	1.99
H1072	Takeda-Shitaka	2.4	2.04	1.98	2.1	2.01
H1072	Seok	1.08	1.98	1.89	1.96	1.97
H1072	DATE	2.71	2.02	1.93	1.92	1.96
H1072	AF2	1.87	2.29	2.26	2.24	2.21
T1032	Baker	0.94	2.62	2.65	2.54	2.23
T1032	Venclovas	2.88	2.06	2.26	2.41	2.44
T1032	Takeda-Shitaka	2.71	2.56	2.35	2.15	1.96
T1032	Seok	1.69	2.6	2.53	2.4	2.35
T1032	DATE	3.54	2.11	1.94	2.14	2.08
T1032	AF2	2.13	4	4.05	3.82	3.95
T1054	Baker	0.87	2.9	2.86	2.91	2.4
T1054	Venclovas	0.83	3.56	3.91	3.9	3.73
T1054	Takeda-Shitaka	3.2	3.78	4.02	3.61	3.79
T1054	Seok	1.73	3.73	3.86	3.93	3.26
T1054	DATE	2.46	3.48	3.88	3.67	3.81
T1054	AF2	1.91	3.92	4	4.29	4.24
T1070	Baker	1.07	3.37	1.95	2.12	2.15
T1070	Venclovas	2.58	4.81	4.54	4.02	3.93
T1070	Takeda-Shitaka	3.64	4.77	4.8	4.71	4.69

T1070	Seok	1.83	4.06	3.47	3.38	3.42
T1070	DATE	3.46	4.68	4.86	4.63	4.31
T1070	AF2	2.72	4.81	4.92	4.94	4.89
T1073	Baker	1.33	3.78	2.98	2.47	2.45
T1073	Venclovas	2.18	3.82	4.41	3.03	4.02
T1073	Takeda-Shitaka	2.99	3.82	4.22	3.92	3.81
T1073	Seok	2.21	3.91	3.58	3.35	3.3
T1073	DATE	2.77	3.9	4.01	4.11	4.11
T1073	AF2	3.06	4.01	4.19	4.29	4.93
T1078	Baker	0.74	3.25	3.01	2.91	2.76
T1078	Venclovas	2.67	2.86	2.32	2.22	2.24
T1078	Takeda-Shitaka	2.57	3.18	2.8	2.6	2.7
T1078	Seok	1.57	2.95	3.13	2.76	2.91
T1078	DATE	3.54	2.71	3.2	2.95	3.07
T1078	AF2	2.64	3.2	3.03	3.13	3.13
T1083	Baker	1.03	2.2	2.18	2.13	2.08
T1083	Venclovas	1.9	2.04	2.29	2.4	2.32
T1083	Takeda-Shitaka	2.3	2.37	2.47	2.28	2.35
T1083	Seok	2.2	2.73	2.67	2.25	2.59
T1083	DATE	1.33	2.12	2.3	2.15	1.91
T1083	AF2	2.44	2.15	2.24	2.15	2.15
T1084	Baker	0.78	2.1	1.96	1.86	1.87
T1084	Venclovas	1.83	2.04	1.86	1.9	1.97
T1084	Takeda-Shitaka	2.71	2.12	2.39	1.87	1.95
T1084	Seok	1.02	2.38	2.31	2.29	2.27

T1084	DATE	1.84	2.38	2.41	2.42	2.73
T1084	AF2	2.41	2.39	2.47	2.62	2.62
Cumulative score		2.068	2.816	2.774	2.667	2.664

D)

**Molprobity of multimeric targets (Single-Sequence)**						
TARGET	Group	Baseline	R_1	R_3	R_6	R_12
H1045	Baker	0.88	2.05	1.97	2.05	1.9
H1045	Venclovas	2.33	3.52	3.42	3.56	3.38
H1045	Takeda-Shitaka	2.85	2.79	2.57	2.65	2.71
H1045	Seok	1.5	2.42	2.4	2.4	2.37
H1045	DATE	2.52	3.09	2.81	2.76	2.74
H1045	AF2	3.9	4.26	4.03	4	4
H1065	Baker	0.9	1.99	1.88	1.97	1.87
H1065	Venclovas	0.76	2.33	2.38	2.55	2.5
H1065	Takeda-Shitaka	2.6	2.35	2.58	2.29	2.64
H1065	Seok	1.4	2.43	2.07	2.23	1.93
H1065	DATE	1.96	2.46	2.45	2.64	2.45
H1065	AF2	3.76	3.33	3.4	3.42	3.4
H1072	Baker	0.81	2.4	2.42	2.67	2.74
H1072	Venclovas	1.89	2.31	2.46	2.25	2.27
H1072	Takeda-Shitaka	2.4	2.9	2.16	2.25	2.26
H1072	Seok	1.08	2.35	2.31	2.18	2.2
H1072	DATE	2.71	3.6	3.54	2.53	2.39
H1072	AF2	2.43	2.66	1.91	2.03	2.06

T1032	Baker	0.94	3.78	3.81	3.66	3.71
T1032	Venclovas	2.88	4.22	4.29	4.67	4.67
T1032	Takeda-Shitaka	2.71	3.76	3.75	3.67	3.62
T1032	Seok	1.69	3.23	3.64	3.67	3.61
T1032	DATE	3.54	3.89	4	3.89	3.98
T1032	AF2	3.5	4.09	4.2	4.16	4.18
T1054	Baker	0.87	3.57	3.58	3.56	3.57
T1054	Venclovas	0.83	4.21	4.1	4.06	4.11
T1054	Takeda-Shitaka	3.2	4.15	3.89	3.99	3.67
T1054	Seok	1.73	3.82	3.98	3.94	3.96
T1054	DATE	2.46	3.78	3.94	3.86	3.83
T1054	AF2	3.8	4.16	4.32	4.1	4.08
T1070	Baker	1.07	4.47	4.63	4.7	4.68
T1070	Venclovas	2.58	4.58	4.52	4.56	4.57
T1070	Takeda-Shitaka	3.64	4.89	4.86	4.79	4.71
T1070	Seok	1.83	4.71	4.88	4.63	4.59
T1070	DATE	3.46	5.04	4.81	4.68	4.56
T1070	AF2	3.69	4.8	4.71	4.78	4.79
T1073	Baker	1.33	4.97	4.85	4.88	4.87
T1073	Venclovas	2.18	4.84	4.89	4.98	4.39
T1073	Takeda-Shitaka	2.99	4.52	4.36	4.8	4.79
T1073	Seok	2.21	4.8	4.48	4.41	4.57
T1073	DATE	2.77	4.97	4.89	4.89	4.89
T1073	AF2	3.11	4.24	4.27	4.38	4.43
T1078	Baker	0.74	3.68	3.5	3.6	3.64

T1078	Venclovas	2.67	3.31	3.71	3.17	3.19
T1078	Takeda-Shitaka	2.57	3.47	3.64	3.5	3.58
T1078	Seok	1.57	3.75	3.81	3.84	3.81
T1078	DATE	3.54	3.93	3.98	3.96	3.52
T1078	AF2	4.13	4.19	4.22	4.23	4.4
T1083	Baker	1.03	2.33	2.11	2.27	2.47
T1083	Venclovas	1.9	2.18	2.03	2.11	2.07
T1083	Takeda-Shitaka	2.3	2.11	2.05	2.01	2.04
T1083	Seok	2.2	2.51	2.49	2.27	2.2
T1083	DATE	1.33	2.77	2.28	2.2	2.19
T1083	AF2	1.92	2.2	1.76	1.79	1.81
T1084	Baker	0.78	2.32	2.26	2.26	2.25
T1084	Venclovas	1.83	2.21	2.24	2.18	2.09
T1084	Takeda-Shitaka	2.71	2.43	2.43	2.36	2.38
T1084	Seok	1.02	2.1	2.16	2.21	2.17
T1084	DATE	1.84	2.19	2.38	2.4	2.42
T1084	AF2	2.19	2.14	2.26	2.22	2.25
Cumulative score		2.199	3.375	3.328	3.312	3.285
